# Functional traits and adaptation of lake microbiomes on the Tibetan Plateau

**DOI:** 10.1186/s40168-024-01979-7

**Published:** 2024-12-20

**Authors:** Xiaoyuan Feng, Peng Xing, Ye Tao, Xiaojun Wang, Qinglong L. Wu, Yongqin Liu, Haiwei Luo

**Affiliations:** 1 Key Laboratory of Lake and Watershed Science for Water Security, Nanjing Institute of Geography and Limnology, Chinese Academy of Sciences, Nanjing, 210008 China; 2 Shenzhen Research Institute, The Chinese University of Hong Kong, Shenzhen, China; 3 Center for Evolution and Conservation Biology, Southern Marine Science and Engineering Guangdong Laboratory (Guangzhou), Guangzhou, China; 4 Center for Pan-Third Pole Environment, Lanzhou University, Lanzhou, China; 5 State Key Laboratory of Tibetan Plateau Earth System, Resources and Environment (TPESRE), Institute of Tibetan Plateau Research, Chinese Academy of Sciences, Beijing, China; 6 Sino-Danish Center for Education and Research, University of Chinese Academy of Sciences, Beijing, China; 7 Simon F. S. Li Marine Science Laboratory, School of Life Sciences and State Key Laboratory of Agrobiotechnology, The Chinese University of Hong Kong, Shatin, Hong Kong SAR, China; 8 Institute of Environment, Energy and Sustainability, The Chinese University of Hong Kong, Shatin, Hong Kong SAR, China

**Keywords:** Tibetan Plateau, Saline lakes, Microbiome, Population genetics, Climate change

## Abstract

**Background:**

Tibetan Plateau is credited as the “Third Pole” after the Arctic and the Antarctic, and lakes there represent a pristine habitat ideal for studying microbial processes under climate change.

**Results:**

Here, we collected 169 samples from 54 lakes including those from the central Tibetan region that was underrepresented previously, grouped them to freshwater, brackish, and saline lakes, and generated a genome atlas of the Tibetan Plateau Lake Microbiome. This genomic atlas comprises 8271 metagenome-assembled genomes featured by having significant phylogenetic and functional novelty. The microbiomes of freshwater lakes are enriched with genes involved in recalcitrant carbon degradation, carbon fixation, and energy transformation, whereas those of saline lakes possess more genes that encode osmolyte transport and synthesis and enable anaerobic metabolism. These distinct metabolic features match well with the geochemical properties including dissolved organic carbon, dissolved oxygen, and salinity that distinguish between these lakes. Population genomic analysis suggests that microbial populations in saline lakes are under stronger functional constraints than those in freshwater lakes. Although microbiomes in the Tibet lakes, particularly the saline lakes, may be subject to changing selective regimes due to ongoing warming, they may also benefit from the drainage reorganization and metapopulation reconnection.

**Conclusions:**

Altogether, the Tibetan Plateau Lake Microbiome atlas serves as a valuable microbial genetic resource for biodiversity conservation and climate research.

Video Abstract

**Supplementary Information:**

The online version contains supplementary material available at 10.1186/s40168-024-01979-7.

## Introduction

The Tibetan Plateau, the highest plateau in the world with an average altitude of over 4500 m, is known as the “Third Pole” of the Earth and as the “Water Tower of Asia” that provides water supplements to billions of people [1]. It contains thousands of lakes which cover a total area of 5 × 10^4^ km^2^ and constitute over half of the total inland water in China [2]. Tibet lakes are a unique habitat featured by high altitude, strong solar radiation, low precipitation, and intense evaporation [3]. This ecosystem is highly sensitive to climate change, exhibiting a regional warming rate of approximately 0.03 °C per year, twice the rate of global warming [4, 5]. Climate warming has accelerated glacial melting and precipitation, leading to the expansion of lake area and the reduction in salinity (desalination) [6], alongside notable changes in drainage patterns particularly in the central Tibetan region [7]. This has also resulted in an increased transport of Pleistocene-aged organic carbon from soils and permafrost to aquatic systems, triggering the mineralization processes that lead to the production of CO_2_ [8]. As a result, the aquatic systems of the Tibetan Plateau have become significant carbon sources, with CO_2_ efflux to the atmosphere having increased by 81% since the 1980s, which in turn strengthens the global warming trend [9].

Compared to low-altitude lakes, the Tibet lakes are less influenced by human activities such as water damming, agricultural runoff, and nutrient pollution from urbanization. This makes Tibet lakes an ideal setting for studying the microbial response to environmental and climate changes [10, 11]. Previous ecological research based on 16S rRNA gene [12–19] and other marker genes [20–22] have revealed that the bacterial communities in the Tibet lakes are primarily regulated by salinity. Despite these insights, metabolic information derived from metagenomic analysis has been available only for a few lake water [23–25] and sediment samples [26, 27]. Not until recently, high-throughput metagenomic sequencing has enabled the establishment of large-scale microbial genome databases, namely Tibetan Plateau Microbial Catalog (TPMC), targeting plateau lakes and other aquatic environments [28, 29]. Nevertheless, a more in-depth comparison of microbial metabolic responses to environmental alteration and climate change remains to be explored. In this study, we conducted metagenomic sequencing of 169 lake water samples from 54 lakes mainly located in the central Tibetan region and covering a wide range of salinity (0.0001–19.1%). Assembly of this new metagenomic dataset led to the Tibetan Plateau Lake Microbiome (TPLM) atlas that comprises 8271 metagenome-assembled genomes (MAGs) with medium or high quality, thus appreciably expanding the microbial genome collection from the aquatic systems in the Tibetan Plateau. We highlight a comparative analysis of population genetic drivers that underly nucleotide diversity between Tibet lakes across a salinity gradient, which is unique compared to the published analyses done with the TPMC dataset, and also include a component of microbial diversity and metabolic potential, which are typically analyzed in other large-scale microbiome studies.

## Materials and methods

All methodological details were described in Supplementary Text. Briefly, water samples were collected from 54 lakes on the Tibetan Plateau between July and September over a 6-year period (2015–2020). Environmental factors and nutrient concentrations for each lake were measured either in the field or in the laboratory. To mitigate potential heterogeneity of microbial communities within a lake, we selected three sites from the same lake including one site approximately 500 m offshore and two closer to the center of the lake, merged the pelagic water samples from the three sites, and performed filtration within a few hours following sample collections. The combined water samples from each lake were initially filtered through 20-μm mesh to remove large particles or eukaryotes, and the filtrates were sequentially filtered through polycarbonate membranes with pore sizes of 3 μm, 0.8 μm, and 0.1 μm to separate patch-associated, intermediate, and free-living microbes, respectively. Microbial DNA was extracted and metagenomic DNA library was constructed from the microbial samples retained on the 3-μm, 0.8-μm, and 0.1-μm filters separately. All libraries were purified with an insert size of 450 bp and subjected to Illumina NovaSeq PE-150 sequencing.

Trimmed reads were assembled using MEGAHIT [30] and metagenomic binning was conducted following the MetaWRAP pipeline [31]. In total, 617 high-quality MAGs (> 90% completeness and < 5% contamination) and 7654 medium-quality MAGs (> 50% completeness and < 10% contamination) following the MIMAG standard [32] were kept for downstream analysis and clustered into species-like operational taxonomic unit (OTU) using dRep [33]. Taxonomic annotation of MAGs was performed using GTDB-Tk with the database version RS202 [34]. The phylogenomic trees were reconstructed based on GTDB alignments using FastTree [35]. The phylogenetic diversity (total branch length spanned by reference genomes in the GTDB database) and gain (additional branch length contributed by TPLM genomes) were calculated accordingly.

The relative abundance of OTUs was then calculated by mapping clean reads to the 2422 species-like representatives using bowtie2 [36]. Genomic features of microbiomes between freshwater lakes, brackish lakes, and saline lakes were estimated for the 2422 genomes each representing a species-like OTU. The value of a genomic feature of the microbiome in each lake sample was calculated as the mean value of the genomic feature across the 2422 OTUs weighted by the relative abundance of each OTU.

Metabolic differentiation in different salinity groups of lakes was predicted by assessing the relative abundance of functional genes. Protein-coding genes were predicted using Prodigal [37], de-replicated using MMseqs2 [38], and functional annotated into KEGG Ortholog (KO). The relative abundance of a KO in a given lake sample was calculated as the sum of the TPM across the 2422 OTUs each multiplied by the corresponding KO copy number within that OTU [39]. Subsequently, the relative abundance of each KO in this sample was normalized by dividing the TPM of the KO by the average TPM of the 27 universal single-copy KOs [40]. Enrichment of KOs between freshwater and saline lakes was identified using Welch’s *t*-test and FDR adjustment.

Taxonomic diversity was assessed at the genus level from metagenomic clean reads based on the 14 marker genes [41] using SingleM. Functional diversity was evaluated based on the relative abundance of KOs mentioned above. Community diversity and correlation to environmental factors were inferred using R package “vegan” and partial Mantel test with 1000 permutations and Bonferroni correction [42]. Enrichment of major phyla in freshwater or saline lakes was identified using STAMP [43].

Intraspecific population genetic parameters, including genome-wide nucleotide diversity (π), single-nucleotide variant (SNV) per Mb, and ratio of nonsynonymous versus synonymous variants (*pN/pS*), were assessed for each species-like OTU in each of the 169 samples using inStrain [44]. These parameters were reanalyzed on fourfold degenerate sites predicted using custom script. The impact of recombination was examined using mcorr [45] based on recruitment results without assembly and using ClonalFrameML [46] based on metagenomic assembled contigs, respectively. The phylogenetic null model analysis was performed following previous study [47].

## Results

### Taxonomic and functional diversity differ between freshwater, brackish, and saline lakes on the Tibetan Plateau

**Fig. 1:**
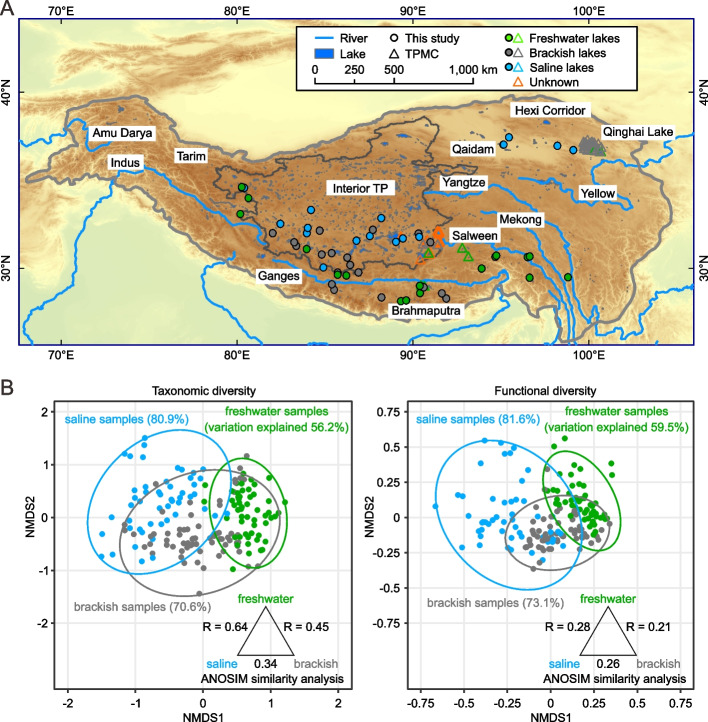
Geographic locations and community structures of the 54 Tibet lakes studied here. Freshwater, brackish, and saline lakes are colored in green, gray, and blue, respectively. **A** Map of the study area. The studied lakes are marked using color dots. Lakes investigated in the Tibetan Plateau Microbial Catalog (TPMC) are marked using open triangles. Large rivers are marked using blue lines. **B** Community variations at taxonomic and functional levels. Non-metric multidimensional scaling analysis (NMDS) plots are derived from the Bray–Curtis distance matrix based on 14 marker genes for taxonomic diversity and 15,943 genes with KEGG annotation for functional diversity. Samples and 95% confidence intervals are shown using color dots and ellipses, respectively. The percentage of variation explained by ecological factors follows each lake type. Community similarities between different types of Tibet lakes are assessed using ANOSIM analysis with significance of 0.001 for all estimations

The 54 investigated Tibet lakes were categorized into three groups according to their salinity [17, 48]: freshwater lakes (salinity < 0.1%), brackish lakes (0.1% < salinity < 3.5%), and saline lakes (salinity > 3.5%; Fig. 1A). This study’s sampling sites encompassed brackish and saline lakes across the central Tibetan region, thereby broadening the scope of recently released Tibetan Plateau Microbial Catalog (TPMC) database [29]. Compared to saline lakes, freshwater lakes exhibited higher dissolved oxygen but lower temperature, pH, cell density, and concentrations of different ions, dissolved organic carbon (DOC), total nitrogen (TN) and phosphorus (TP), and dissolved inorganic nitrogen and phosphorus (*p* < 0.001, Welch’s *t*-test; Fig. S1 and Table S1). The taxonomic analysis based on the 14 marker genes [41] showed that Actinobacteriota is the most dominant phylum in the Tibet lakes (35%), followed by γ-Proteobacteria (13%), Cyanobacteria (11%), α-Proteobacteria (11%), β-Proteobacteria (8%), Firmicutes (6%), Halobacterota (4%), Bacteroidota (3%), Verrucomicrobiota (2%), and Planctomycetota (1%; Fig. S2A). Among these, Cyanobacteria, α-Proteobacteria, and β-Proteobacteria were enriched in freshwater lakes, whereas γ-Proteobacteria and Halobacterota were enriched in saline lakes (*p* < 0.01, Welch’s *t*-test). Archaea excluding Halobacterota constituted only 0.2% of the total prokaryotic community in Tibetan lakes.

The Shannon index at both taxonomic level inferred based on the 14 marker genes [41] and functional level inferred based on 15,943 functional genes (Table S2) was significantly higher in freshwater lakes than that in saline lakes (*p* < 0.001, Welch’s *t*-test; Fig. S2B), consistent with earlier reports showing a decreasing taxonomic diversity with increasing salinity based on 16S rRNA gene amplicon sequences [12, 13]. The community structure based on Bray–Curtis measures supported a clear separation between freshwater and saline lakes at the taxonomic level (ANOSIM R of 0.64, *p* = 0.001) and a moderate separation at the functional level (ANOSIM R of 0.28, *p* = 0.001), both of which were transitioned and bridged by brackish lakes (Fig. 1B), with the caveat that the potential within-lake variation in community composition across time and locations was not considered. As a negative control, community compositions across different pore sizes were well mixed (ANOSIM R < 0.08, *p* > 0.05; Fig. S3). These results demonstrated a crucial role of salinity in structuring the microbial communities across the Tibet lakes.

To identify factors that shape the community structure, we correlated taxonomic and functional diversity to the 27 measured ecological factors (Fig. S4). Across all lakes, salinity, conductivity, and concentrations of sodium and potassium were among the most important drivers of both taxonomic (*R*^2^ of 0.40, 0.41, 0.34, 0.28, respectively; Bonferroni adjusted *p* < 0.01, applicable below unless otherwise noted) and functional community structures (*R*^2^ of 0.62, 0.64, 0.73, and 0.56, respectively), though these factors were mutually correlated. Likewise, the nutrient measures including TN, TP, ammonium, and phosphate, which were also mutually correlated, showed high positive correlation with the community structure at both taxonomic (*R*^2^ varied from 0.13 to 0.29) and functional levels (*R*^2^ varied from 0.47 to 0.59). Temperature and dissolved oxygen were also important drivers of the community structure (*R*^2^ of 0.28 and 0.26 at taxonomic level and R^2^ of 0.44 and 0.39 at functional level). Performing these analyses separately for each salinity group of the lakes, we showed that many of these patterns were conserved but faded in saline lakes and brackish lakes, whereas they were completely missing in freshwater lakes (Fig. S4). The weak correlations between environmental factors and community structure across freshwater lakes are consistent with the low explanatory power of ecological factors across freshwater lakes (56.2% at the taxonomic level and 59.5% at the functional level) compared to across brackish lakes (70.6% and 73.1%) and across saline lakes (80.9% and 81.6%; Fig. 1B). This pattern cannot be fully ascribed to the reduced variations of the physiochemical parameters in freshwater lakes such as salinity, since many factors such as temperature, dissolved oxygen, and TN displayed large variations (Fig. S1).

### A microbial genome atlas *of Tibet* lakes based on metagenome-assembled genomes

**Fig. 2:**
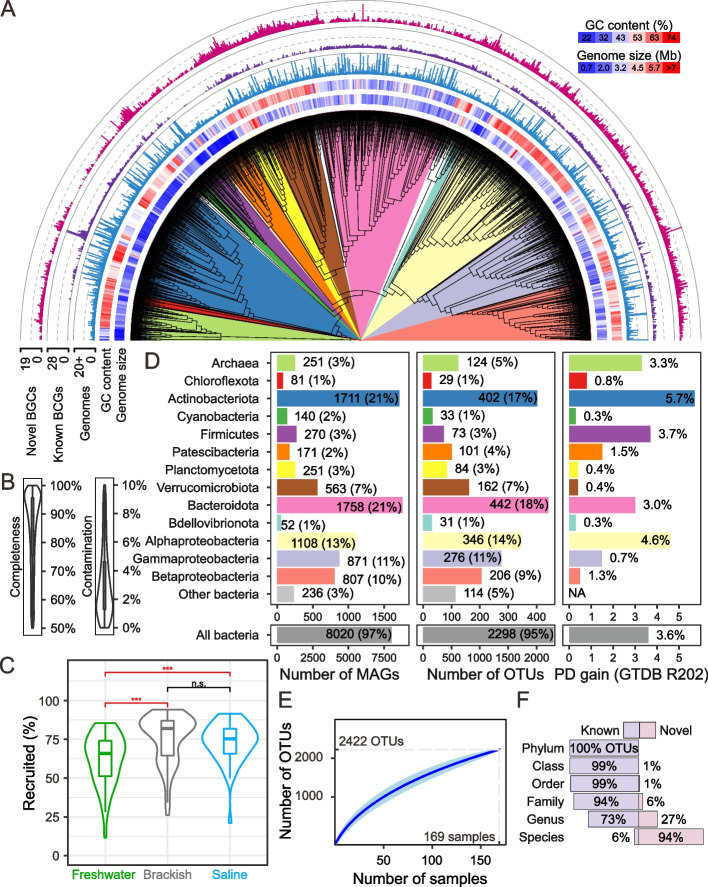
The Tibetan Plateau Lake Microbiome (TPLM) atlas. **A** Phylogenetic tree for 2422 species-like OTUs. The maximum likelihood tree is built based on GTDB alignments of 120 and 122 single-copy genes universally distributed in bacteria and archaea, respectively, using IQ-Tree with LG + I + G model. Archaeal and bacterial trees are combined manually and branch length is ignored for better visualization. The major phyla and classes of Proteobacteria are marked with different colors. The blue-to-red heatmaps represent estimated genome size (defined as assembled genome size divided by the sum of genome completeness and contamination) and GC content. The strip charts from inner to outer circle represent number of genomes affiliated with a species-like OTU, number of known BGCs, and number of novel BGCs, respectively. **B** Quality of the 8271 TPLM MAGs. **C** Recruitment rates of metagenomic sequencing reads against the 8271 TPLM MAGs. The *p* values of < 0.001 based on Welch’s *t*-test between different types of lakes are colored in red. Abbreviation: n.s. not significant, *** *p* < 0.001. **D** Taxonomic assignment and novelty. The taxonomic assignment is performed using GTDB-Tk with the database version of RS202. The phylogenetic diversity (PD) gain is represented as the difference between the total branch lengths of GTDB reference genomes (RS202) with and without the addition of the 8271 TPLM MAGs. **E** Rarefaction curve of species-like OTUs detected in lake samples. **F** Proportion of species-like OTUs annotated in GTDB database at each taxonomic level

The Tibetan Plateau Lake Microbiome (TPLM) genome atlas was generated from collected lake samples using shotgun metagenomic sequencing with an average depth of 110 Gb per sample (18 Tb in total). Filters with pore sizes of 3 μm, 0.8 μm, and 0.1 μm were assembled and binned separately, except for eight lake samples with insufficient DNA for metagenomic sequencing (Table S1). We recovered 8271 MAGs that meet the minimum requirement of medium quality according to the MIMAG standard (i.e., completeness > 50% and contamination < 10%; Fig. 2A and Table S3) [32]. These MAGs had average completeness of 84.5% (± 13.8%) and average contamination of 3.2% (± 2.5%; Fig. 2B). The estimated genome size of these MAGs spanned from 0.6 Mb for unclassified Mycoplasmatales to 13.9 Mb for the family UBA1268 in Planctomycetes, and their GC content varied from 22 to 74% (Table S3). Interestingly, the average estimated genome size of TPLM genomes (~ 3.14 Mb) was significantly smaller than those from freshwater and saline lakes in the recently released TPMC database (~ 3.30 Mb; *p* < 0.01, Welch’s *t*-test) [29], which may be attributable to either different methodologies or distinct sampling regions (Fig. 1A), or a combination of both. The TPLM MAGs recruited an average of 70.7% (± 16.2%) of the metagenomic reads (Fig. 2C), suggesting that our TPLM genome atlas covered a major fraction of the TPLM diversity. We note that the freshwater lake microbiomes (62.3% ± 16.3%) had a significantly lower metagenomic recruitment rate compared to brackish and saline lake microbiomes (75.6% ± 15.7% and 73.0% ± 12.8%, respectively; *p* < 0.001, Welch’s *t*-test). This may result from the greater biodiversity in freshwater lakes (Fig. S2B).

The 8271 MAGs were clustered into 2422 species-like operational taxonomic units (OTUs) based on whole-genome average nucleotide identity (ANI) of 95% (Fig. 2D and Table S4), a threshold widely used to define species boundaries [49]. The number of species-like OTUs steadily increased with the number of samples (Fig. 2E), indicating the presence of yet undiscovered microbial lineages. These MAGs further represented 50 known phyla, 90 classes, 211 orders, and 356 families based on the GTDB classification (RS202; Table S4). The TPLM atlas provided substantial genomic novelty as they increased archaeal and bacterial phylogenetic diversity by 3.6% and 3.3%, respectively, compared to the reference genomes in the GTDB database (RS202; Fig. 2D). When TPMC reference genomes were additionally considered, the TPLM genomes still enhanced archaeal and bacterial phylogenetic diversity by 1.9% and 1.2%, respectively (Fig. S5A). There were 2265 (93.5%) species-like OTUs that were not assigned to any known species, therefore representing potential novel species (Fig. 2F). They made 650 (26.8%) novel genera and 142 (5.9%) novel families, thereby appreciably expanding the known diversity of microbes. Furthermore, 1377 of the 2422 TPLM species-like OTUs (56.9%) exhibited an ANI below 95% relative to the TPMC genomes (Fig. S5B), further illustrating that novel genetic resources were harvested in the TPLM atlas compared to the existing TPMC database.

We further selected a representative MAG with highest estimated quality (defined as completeness minus five times of contamination [50]) from each of the 2422 species-like OTUs and predicted 7969 putative secondary metabolite biosynthetic gene clusters (BGCs), among which 5088 (63.8%) may produce novel secondary metabolites (Fig. 2A, Fig. S6A, and Table S5). Among the novel BGCs, 794 formed complete clusters and 306 had lengths exceeding 30 Kb. Alphaproteobacteria were the most promising sources of new BGCs, and terpenes constituted the largest fraction (41%) of novel products (Fig. S6A). Together, these results highlighted the significant biosynthetic potential and functional novelty of the Tibetan Plateau microbiomes. Intriguingly, some BGC categories synthesizing potential antimicrobial compounds displayed distinct distribution patterns in Tibet lakes. For example, bacteriocin cluster and non-ribosomal peptide synthetase (NRPS) were enriched in freshwater lakes, while betalactone clusters were enriched in saline lakes (FDR adjusted *p* < 0.01; Fig. S6B), indicating that microbes in different salinity groups of lakes may engage in community competition by employing diverse antibiotics [51].

Next, we compared the genomic features between freshwater and saline lake microbiomes (Fig. S7) by weighting the genomic features of 2422 representative genomes according to their relative abundance in different lakes. Saline lake microbiomes had a lower number of genes encoding carbohydrate-active enzymes (CAZY) per Mb (28.3 ± 5.4) and a higher optimal growth temperature (29.1 ± 2.7 °C) than freshwater lake microbiomes (31.6 ± 3.2 copies per Mb and 27.3 ± 0.9 °C; *p* < 0.001), which is consistent with a higher organic carbon load in freshwater lakes [48, 52] and higher in situ temperature in saline lakes (Fig. S1). Saline lake microbiomes further exhibited slightly smaller estimated genome size (2.46 ± 0.56 Mb) and higher GC content (55.4 ± 4.8%) compared to freshwater lake microbiomes (2.71 ± 0.55 Mb and 54.2 ± 4.3%; *p* < 0.05). No significant difference was identified in terms of coding density and the number of carbon (C-ARSC) and nitrogen (N-ARSC) atoms per residue side chain.

### Microbial processes differ between freshwater, brackish, and saline lakes

**Fig. 3:**
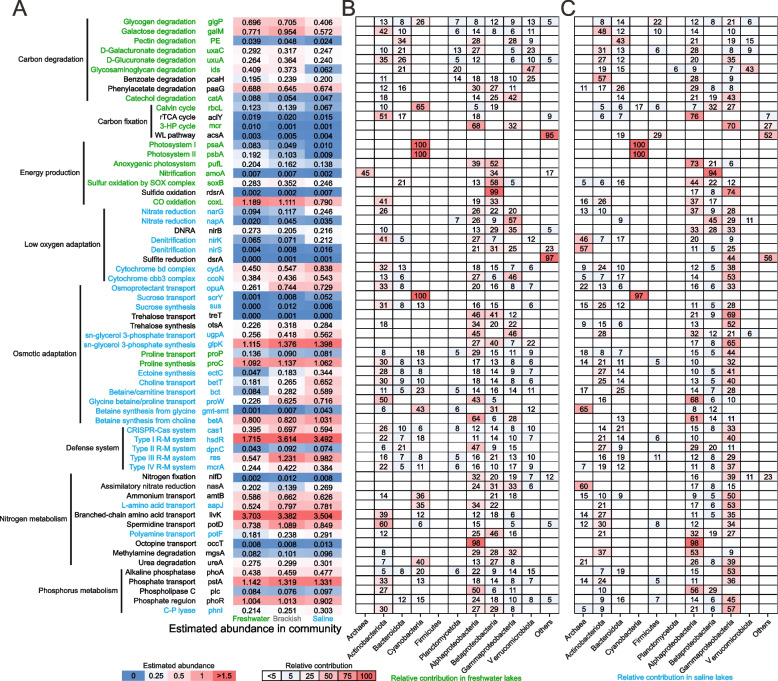
Metabolic comparison of selected functional genes. **A** Metabolic comparison between freshwater lakes, brackish lakes, and saline lakes on the Tibetan Plateau. The relative abundance of selected functional genes is normalized using the average TPM of 27 universal single-copy KOs and is shown using a heatmap with a color gradient. Gene functions and names are shown on the left panel, and their relative abundance in lake samples is shown using a heatmap with a blue-to-red color gradient. Gene enrichment analysis is performed between freshwater and saline lakes using STAMP, and gene enriched in freshwater and saline lake microbiomes (FDR adjusted *p* value < 0.01) are colored in green and blue, respectively. Genes without enrichment patterns are colored in black. The gene *rbcL*, *amoA*, *amtB*, *dsrA*, and *rdsrA* refer to type I/II *rbcL* for Calvin cycle (Fig. S8A), copper membrane monooxygenases specific for ammonia oxidation (Fig. S8B), high-affinity ammonium transporter (Fig. S8C), reductive *dsrA* for sulfite reduction, and oxidative *dsrA* for sulfide oxidation, respectively (Fig. S8D). DNRA, dissimilatory nitrate reduction to ammonium. **B**, **C** Relative contribution of different taxonomic groups in freshwater lake (**B**) and saline lake microbiomes (**C**). The proportions of relative abundance among eleven taxonomic groups are shown using a heatmap with a white-to-red color gradient. Proportions of > 5% are given with actual numbers for better visualization. Specifically, three putative *amoA* genes are classified as β-Proteobacteria-type *amoA*, while the corresponding MAGs are affiliated with γ-Proteobacteria and Firmicutes (Fig. S8B). These genes are located on short contigs with lengths of 2.3, 3.3, and 7.8 Kb, respectively, and may be sourced from binning errors. Thus, these genes are considered to belong to β-Proteobacteria in the heatmap plot

A few key genes of the ecologically relevant metabolic pathways showed differential enrichment between freshwater lakes and saline lakes (Fig. 3). Freshwater lake microbiomes were enriched with genes involved in the degradation of various carbohydrates and aromatic compounds, in line with the more abundant CAZY genes in them (Fig. S7). Notably, some of these compounds, such as pectin, d-galacturonate, and d-glucuronate, are major components of plant cell walls [53, 54]. These findings suggest that freshwater lake microbiota has evolved metabolic capability to utilize a broader range of organic carbon, particularly those from terrestrial input.

Calvin cycle is the most prevalent pathway for C fixation used by Cyanobacteria and many aerobic chemolithotrophs [55]. Indeed, it was the primary C-fixing pathway in the TPLM and more common in freshwater lake microbiomes than in saline lake microbiomes (Fig. 3A). Intriguingly, the relative abundance of its marker genes type I/II *rbcL* (Fig. S8A) was significantly and positively correlated with photosystem marker genes *psaA* (photosystem I) and *psbA* (photosystem II) in freshwater lakes (*R*^2^ = 0.66 and 0.58), but not in saline lakes (*R*^2^ = 0.07 and 0.06; Fig. S9), suggesting a shift in the functional group of C fixation from photoautotrophs (primarily Cyanobacteria; Fig. 3B) in freshwater lakes to chemoautotrophs (primarily Proteobacteria; Fig. 3C) in saline lakes [42].

Phototrophy and chemolithotrophy allow microbes to conserve energy through acquisition of light and oxidation of inorganic compounds, respectively. Our analysis showed that freshwater lake microbiomes were enriched with both phototrophic and chemolithotrophic pathways compared to saline lakes. The former included *psaA* and *psbA* for oxygenic photosynthesis by Cyanobacteria and *pufL* for aerobic anoxygenic photosynthesis primarily by α- and β-Proteobacteria, and the latter included *amoA* for nitrification (primarily Thaumarchaea and β-Proteobacteria), *sox* for sulfur oxidation (Bacteroidota, α-, β- and γ-Proteobacteria), and *cox* for carbon monoxide oxidation (Actinobacteria, α- and β-Proteobacteria). This suggests that freshwater lake microbiota faced greater energy limitation than saline lake microbiota.

Saline lake microbiomes were enriched with pathways that lead to energy conservation when oxygen becomes limited. For example, genes related to nitrate reduction and denitrification, which use nitrate and nitrite as alternative electron acceptors, were enriched in saline lake microbiomes. Both *bd-* and *cbb3-*type cytochromes with high oxygen affinity were more commonly found in saline lake microbiomes. Thus, the differential distribution patterns of genes encoding membrane-associated reductases reflect microbial adaptation to freshwater and saline lakes with higher and lower concentrations of dissolved oxygen, respectively.

Saline and freshwater lakes had differential distribution of the osmolyte transport and synthesis genes. For example, saline lake microbiomes carried more genes responsible for the transport and synthesis of many classical osmolytes, such as sugars (sucrose), polyols (sn-glycerol 3-phosphate), amino acid derivatives (ectoine), and quaternary amines (choline, glycine betaine, carnitine), whereas freshwater lake microbiomes were enriched with proline transport and synthesis genes. Proline is multifunctional and may be alternatively used for protein synthesis and/or act as a signaling molecule [56].

Tibet lakes harbor abundant and novel viruses [57]. Microbes in saline lakes were enriched with genes related to defense mechanisms, such as the CRISPR-Cas system and various restriction-modification (R-M) systems. The prevalence of these genes across multiple microbial lineages (e.g., Actinobacteriota, Bacteroidota, α-, β- and γ-Proteobacteria) suggests that they may provide a competitive advantage in counteracting the intense viral predation that typically occurs in saline lake ecosystems [58].

While there were important differences of nitrogen (N) and phosphorus (P) concentrations between freshwater and saline lakes (Fig. S1), most genes involved in N and P acquisition were not differentially distributed. For example, genes for N fixation, nitrate assimilation, and the transport and utilization of organic N compounds were equally distributed between freshwater and saline lakes, though transporters for L-amino acid and polyamine were enriched in saline lakes (Fig. 3A). Likewise, most pathways for P scavenging or cost minimization including alkaline phosphatase, high-affinity phosphate transporter, lipid remodeling under P limitation, and the two-component signal transduction system responding P limitation were not differentially distributed between freshwater and saline lake microbiomes, though genes for C-P lyase were enriched in saline lake microbiomes (Fig. 3A). Since ammonium and phosphate are in much higher concentrations in saline lakes compared to those in freshwater lakes (Fig. S1), the few pathways enriched in saline lakes were less likely used to compensate for nutrient scarcity.

### Saline lake microbiota are under more effective purifying selection than freshwater microbiota on the Tibetan Plateau

**Fig. 4:**
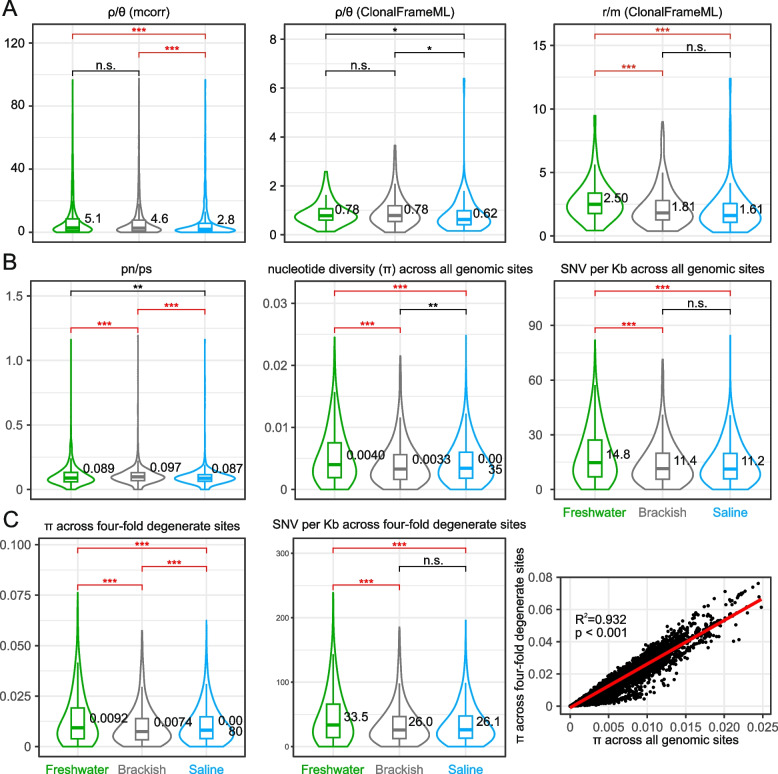
Comparisons of population genetic parameters. These parameters are estimated for each species-like OTU in each lake sample separately. Freshwater, brackish, and saline lakes are colored in green, gray, and blue, respectively, with their median value shown in the violin plots for better visualization. The *p* values of < 0.001 based on Welch’s *t*-test between different types of lakes are colored in red. **A** Microbial recombination. The relative rate (*ρ/θ*) and effect (*r/m*) of recombination to mutation are estimated using two methods. The *ρ/θ* value is first calculated based on metagenomic recruitment using mcorr, and the *ρ/θ* and *r/m* values are then calculated based on assembled contigs using ClonalFrameML. **B** Intraspecific genetic diversity calculated based on metagenomic recruitment using inStrain. A smaller *pN/pS* value generally represents a more effective purifying selection. **C** Neutral genetic diversity on fourfold degenerate sites. Abbreviation: n.s. not significant, *p < 0.05, **p < 0.01, ***p < 0.001

We leveraged 2242 species-like OTUs to determine several intraspecific population genetic parameters (Fig. 4). First, we estimated the relative rate (*ρ/θ*) and effect (*r/m*) of recombination to mutation [45]. Both *ρ/θ* and *r/m* were significantly greater in freshwater lake microbiomes than in saline lake microbiomes (*p* < 0.05, Welch’s *t*-test; Fig. 4A), suggesting that recombination had a greater role in shaping the former than the latter. The difference cannot be attributed to differential opportunities for physical contacts between cells required for several mechanisms of recombination such as conjugation, as cell density was much lower in freshwater lakes (Fig. S1 and Table S1). Instead, this observation could be linked to the enrichment of defense systems in saline lake microbiomes (Fig. 3A). Additionally, we tested the effectiveness of natural selection using the ratio of population-level nonsynonymous versus synonymous variants (*pN/pS*) as a proxy [44]. Since synonymous polymorphisms are largely neutral and nonsynonymous changes are more likely to be deleterious [59], a *pN/pS* ratio of < 1 indicates purifying selection acting to eliminate deleterious variants. The genome-wide *pN/pS* value was found to be well below one for most species-like OTUs in most samples and was significantly lower in saline lake populations compared to that in freshwater and brackish lake populations (*p* < 0.01, Welch’s *t*-test; Fig. 4B), indicating that purifying selection is in general more effective in saline lakes.

Next, we showed a greater intraspecific genetic diversity at neutral sites in freshwater lake populations than brackish and saline lake populations, as shown by more single-nucleotide variants (SNVs) per Kb and greater nucleotide diversity (π) in freshwater lake microbiomes (Fig. 4C). We used fourfold degenerate sites as a proxy for neutral sites because nucleotide mutations at these sites are always synonymous (silent) and immune from selective pressure imposed at protein sequence level. According to theory, genetic diversity at these sites scales positively with effective population size (*N*_*e*_) defined as the size of an ideal population that harbors the same amount of neutral genetic diversity as the actual population [60, 61]. The trend remained when all nucleotide sites in the genome were used (Fig. 4B) due to strong correlation between nucleotide diversity across all sites and fourfold degenerate sites (*R*^2^ = 0.93, Fig. 4C).

## Discussion

In this study, we present a comprehensive TPLM genome atlas from across 54 Tibetan lakes that are geographically widely distributed, with a focus on the lakes from the central Tibetan region. This TPLM atlas complements and expands the TPMC database, which was built on ecologically diverse samples (lakes, rivers, hot springs, wetlands, and glaciers), with its lake samples collected from only four localized areas (Lhasa, Nagqu, Shannan, and Qinghai Lake) [29]. Although the TPLM atlas contains fewer species-like OTUs than the TPMC database (2422 vs. 10,723), TPLM genomes increase the known archaeal and bacterial phylogenetic diversity by 1.9% and 1.2%, respectively, from the TPMC and GTDB genome databases (Fig. 2D).

On the Tibetan Plateau, freshwater lakes are more limited in carbon and energy supplies than saline lakes (Fig. S1) [17, 62, 63]. To facilitate adaptation in the more oligotrophic freshwater lakes, freshwater microbiota had more genes involved in carbon fixation and energy conservation through photosynthesis and oxidation of reduced inorganic compounds and more genes for the utilization of recalcitrant terrestrial DOM [48, 52, 64]. In contrast, microbiota in the saline lakes is featured by carrying more genes encoding the transport and synthesis of osmolytes and more genes for anaerobic metabolism, which enable them to adapt to a wider range of salinity and lower levels of dissolved oxygen. Freshwater lakes, together with brackish lakes, may hold greater ecological significance in context of warming climate, as the plateau lakes have been experiencing declining salinity due to increased precipitation and enhanced glacier melting [6]. As a result, the plateau lake microbes such as phototrophs and chemolithotrophs, which are generally better adapted to oligotrophic freshwater environments but outcompeted in saline lakes where they are stressed by increased osmotic pressure and decreased CO_2_ availability [65, 66], may thus benefit from the ongoing lake desalination process, thereby playing increasingly important roles in elemental cycling and energy transformation in the Tibet lake ecosystems.

While N and P concentrations (Fig. S1) are lower in freshwater lakes compared to those in saline lakes, pathways leading to genomic adaptation to nutrient limitation were not observed. For example, N limitation is expected to drive the reduction of genome size, GC content, and N-ARSC in oligotrophic environments such as the pelagic ocean [67, 68], but these features were not observed in the freshwater lake MAGs relative to saline lake MAGs (Fig. S7). Moreover, most genes involved in N acquisition were equally distributed across the plateau lake microbiomes regardless of the N concentrations. This is likely because most studied plateau lakes have high levels of dissolved inorganic nitrogen (DIN; 9.5 ± 0.3 μM in freshwater lakes, 38 ± 52 μM in brackish lakes, and 71 ± 91 μM in saline lakes; Table S1) compared to the surface water of oligotrophic oceans where N is very scarce (3.3 ± 4.9 μM) [69]. Likewise, the P scavenging or cost-minimizing genes were not more abundant in freshwater lakes where P is at lower levels compared to saline lakes, matching well with the geochemical pattern that the phosphate concentrations were much higher in most of the studied plateau lakes (e.g., 0.27 ± 1.05 μM in freshwater lakes, 8.7 ± 18.3 μM in brackish lakes, and 45 ± 98 μM in saline lakes) compared to subtropical ocean regions with severe P limitation such as the Sargasso Sea (< 10 nM) [70] and the Eastern Mediterranean (< 2 nM) [71].

The reduced *pN/pS* of microbial populations in saline lakes compared to that in freshwater lakes suggests that selection is in general more effective in the former than in the latter (Fig. 4B). In theory, several factors could contribute to this, including larger effective population size (*N*_*e*_), higher recombination rate, and stronger strength of selection (i.e., greater selection coefficient, *s*). However, neither* N*_*e*_ (approximated by nucleotide diversity at fourfold degenerate sites) nor recombination accounts for the observed difference in selection effectiveness, as both factors exhibit lower levels in saline lake populations (Fig. 4A, C). We therefore argue that the difference in the strength of selection imposed by the lake environments may explain the observed difference in the effectiveness of selection. Saline lakes represent harsher environments where microbial inhabitants are under greater selective pressures compared to freshwater lakes. Hence, mutations arising from the saline lakes may be more deleterious and are thus subjected to greater functional constraints than similar mutations occurring to microbes in the freshwater lakes [66]. Indeed, greater strength of selection has been reported in other extreme habitats including thermal [72] or acidic [73] environments compared to temperate and neutral habitats, respectively. It is therefore reasonable to hypothesize that the ongoing lake desalination in a warming climate may have already been driving the relaxation of the selective pressure on the microbial populations in saline lakes, enabling the microbial inhabitants to be more tolerant to deleterious mutations.

In most comparisons of the physiochemical properties, the freshwater lakes and the saline lakes represent the two extremes of the Tibet lakes, whereas the brackish lakes fall in between (Fig. S1). Accordingly, the microbial community structure of freshwater lakes and saline lakes are well separated at both taxonomic and functional levels but become connected through the microbiota of brackish lakes (Fig. S1 and Table S1). Notably, the brackish lakes appear to be home to microbial populations that have smaller *N*_*e*_ compared to the freshwater and saline lakes, as evidenced by their microbial populations associated with the smallest nucleotide diversity at fourfold degenerate sites (Fig. 4C) and largest *pN/pS* ratio (Fig. 4B). Many processes are known to affect the magnitude of *N*_*e*_, including, but not limited to, demographic history, genetic linkage, and population subdivision [74], but those discussions were largely based on studies of higher eukaryotic species. In marine bacterioplankton, an emerging pattern was noted in which genome-reduced lineages have smaller *N*_*e*_ than lineages with large and variable genomes [75, 76]. Important processes that likely drive their magnitude differences of *N*_*e*_ include the frequency of selective sweeps, the strength of clonal interference (i.e., beneficial mutations arising from different loci compete for fixation), the rate of recombination, and the formation of metapopulation structure [61]. It is interesting to find that in the Tibetan Plateau, microbial populations in brackish lakes may have smaller *N*_*e*_ than those in freshwater and saline lakes, but the processes leading to this remarkable pattern is not clear. It is worth mentioning that our analyses were based on metagenomic sequences. However, MAGs are not ideal for population genetic analysis; MAGs are largely missing genetic linkage information which is crucial for population genetic inferences, and they do not allow distinguishing pre-existing genetic variants from newly arising mutations [77, 78]. Additional insights into the evolutionary drivers of Tibetan lake microbiota may be gained through future population genomic studies based on large-scale closely related isolates.

## Concluding remarks

In this study, we present the TPLM genomic atlas for Tibetan lakes, which span a broad salinity gradient and a wide geographical distribution. We find that community structures, nutritional strategies, and evolutionary drivers of freshwater lake microbiomes differ from those of saline lake microbiomes. Under an increasingly warming climate, the Tibet lakes are experiencing area expansion and desalination due to intensified precipitation and glacier melting [6]. On one hand, this will deliver more terrestrial organic matter to the lakes and subsequently release more CO_2_ from the lakes, thereby providing a positive feedback on climate warming [9]. On the other hand, lake desalination will promote oxygenic phototrophs and aerobic chemolithotrophs that fix CO_2_ and inhibit anaerobic respiration including denitrification that releases the greenhouse gas nitrous oxide, thereby exerting negative feedback. Such a negative feedback loop parallels the reported rise in primary production by plants across the Tibetan Plateau [79] and by phytoplankton within polar oceans [80] in context of global warming.

While lake desalination may make suitable habitats for halophiles (high salinity-loving organisms) less available, the hydrological drainage reorganization as a result of climate warming, glacial melting, and increased precipitation could enhance lake connectivity, especially in the central Tibetan region [7] where most sampled saline lakes are located. This process will unite locally isolated populations to form genetically connected metapopulations, thereby diminishing the impact of dispersal limitation, which is the main process that shapes the community assembly in Tibet lakes (Fig. S10) as shown in a null model analysis [47], and increasing *N*_*e*_ of many microbial species. A larger *N*_*e*_ increases the efficiency of natural selection that acts to eliminate deleterious mutations and promote beneficial mutations, thus conferring them a greater potential to adapt in a changing environment [75, 81]. While quantifying these competing processes that drive microbial evolution [82] and feedback on biogeochemical cycling is beyond the scope of the present study, we anticipate that the TPLM genome atlas generated here, together with previously established large-scale genome databases, will continue to be a valuable resource to understand microbial processes under climate change and their roles in climate regulation.

## Supplementary Information

Supplementary Material 1.

Supplementary Material 2.

## Data Availability

The clean reads, assembled contigs, and MAGs are available at the NODE (https://www.biosino.org/node/) and NCBI databases under the accession number OEP004147 and PRJNA982183, respectively.
