# Supplementary material for: Functional traits and adaptation of lake microbiomes on the Tibetan Plateau
Source: Microbiome. 2024 Dec 20;12:264. doi: 10.1186/s40168-024-01979-7 (PMC11662823; doi:10.1186/s40168-024-01979-7)

Supplementary Information for

Functional traits and adaptation of lake microbiomes on the Tibetan Plateau

Xiaoyuan Feng, Peng Xing, Ye Tao, Xiaojun Wang, Qinglong L. Wu, Yongqin Liu, Haiwei Luo

This file includes:

Supplementary Text: Materials and Methods

Supplementary References

Supplementary Figures

Supplementary Methods

<u>Study sites and environmental metadata measurement</u>

Water samples were collected from 54 lakes on the Tibetan Plateau between July and September over a six-year period (2015-2020). To mitigate potential heterogeneity of microbial communities within a lake, we selected three sites from the same lake including one site approximately 500 meters offshore and two closer to the center of the lake, merged the pelagic water samples from the three sites, and performed filtration within a few hours following sample collections. The pelagic water from three sites was subsequently combined for filtration within a few hours after sampling. In particular, water depth was measured using a Speedtech SM-5A portable echo sounder at each site and then divided into five equal parts from the surface to the bottom on the spot. Lake water was collected from each depth by using a 5-L Niskin bottle and mixed in a 25L polycarbonate carboy to obtain the water sample for each site. Water collected at the three sites were subsequently mixed in equal volumes and approximately 10-15 L of water was pre-filtered for each lake through a 20 μm mesh to remove large particles and eukaryotes. The filtrate was sequentially filtered through polycarbonate membranes (Millipore) with pore sizes of 3 μm, 0.8 μm, and 0.1 μm, to separate patch-associated, intermediate, and free-living microbes, respectively. Filters for each pore size were fast-frozen in liquid nitrogen. Water temperature, conductivity, salinity, pH, and dissolved oxygen (DO) concentration were logged at 0.5m interval in the field using a 6600 Multi-Parameter Water Quality Sonde (YSI Inc., Yellow Springs, Ohio), and the mean value across the

collected samples was used to represent the environmental factor of a lake.

The concentrations of total phosphorus (TP), total nitrogen (TN), dissolved organic carbon (DOC), ammonium (NH_4-N), nitrate (NO_3-N), nitrite (NO_2-N), and soluble phosphorus (PO_4-P) were measured using standard methods (APHA/AWWA/WEF 2017 (1)). Additionally, 10 mL water from each lake was fixed with glutaraldehyde (final concentration 2% v/v) for the flow-cytometric enumeration of total bacterial cells following a previously established protocol (2). The total dissolved boron (B), barium (Ba), manganese (Mn), calcium (Ca), magnesium (Mg), sodium (Na), potassium (K), silicon (Si), and strontium (Sr) were measured using inductively coupled plasma optical emission spectrometry (ICP-OES). The concentrations of chloride (Cl) and sulfate (SO_4) were determined by ion chromatography. The lakes were categorized into three groups based on their salinity: freshwater lakes (salinity <0.1%, n = 17), brackish lakes (0.1%< salinity <3.5%, n = 19), and saline lakes (salinity >3.5%, n = 18). Statistical analysis of environmental factors was performed using R package 'ggpubr' v0.4.0 (https://rpkgs.datanovia.com/ggpubr).

DNA extraction, and sequencing

Microbial DNA was extracted from 3 μm, 0.8 μm, and 0.1 μm filters separately using DNA extraction kit (E.Z.N.A.® Water DNA Kit, Omega Biotek, USA). Metagenomic DNA library was constructed for each sample using the Illumina TruSeq DNA Sample Prep Kit following the manufacturer's instructions, which requires a minimal DNA amount of 100 ng. However, the collected genomic DNAs

from eight lakes did not meet this threshold, and DNAs from all three pore sizes were therefore combined to construct one library for each of these lakes. All libraries were purified with an insert size of 450 bp using Ampure XP beads (Beckman-Coulter) and then subjected to Illumina NovaSeq PE-150 sequencing.

Metagenomic assembly, binning, and dereplication

Sequencing raw reads were quality trimmed using Trimmomatic v0.33 (3) with options ‘SLIDINGWINDOW:4:15 MINLEN:75’ and mapped against human HG19 sequences using the mem module in BWA v0.7.17-r1188 (4) to remove potential human contaminations (5). Clean reads from each sample were assembled separately to retain natural sequence variations across samples using MEGAHIT v1.1.1 (6) with options ‘--min-contig-len 500’.

The sequencing depth for each assembled contig was determined by mapping reads from the corresponding sample using the ‘jgi_summarize_bam_contig_depths’ script in bbMAP package v38.79 (https://sourceforge.net/projects/bbmap). Binning was conducted following the MetaWRAP pipeline v1.0.2 (7) with default parameters by MetaBAT2 (8), CONCOCT (9), and MaxBin (10). Preliminary bins were dereplicated and aggregated using DAS_Tool v1.1.1 (11). Completeness and contamination of metagenome-assembled genomes (MAGs) were estimated using CheckM v1.1.3 (12). In total, 8,271 MAGs with medium- or high-quality following the MIMAG standard (>50% completeness and <10% contamination (13)) were kept for downstream analysis.

The clustering of the 8,271 sequenced MAGs was performed using dRep v3.2.0

(14) with option '-pa 0.95', which sets the average nucleotide identity at 95% for species-like OTU clustering (15). MAGs with higher estimated quality (defined as completeness minus five times of contamination (16)) were chosen as representatives for each species-like OTU.

Gene annotation, clustering, and tree construction

Protein-coding genes were predicted using Prodigal v2.6.3 (17) with option '-p meta' and de-replicated using MMseqs2 v13.45111 (18) with options '-k 80 -min-seq-id 0.8', which sets *k*-mer length at 80 and average nucleotide identity at 80% for gene clustering. Functions of de-replicated genes were annotated by EggNOG with EggNOG-mapper v2.0.1 (19), Carbohydrate-Active enZYmes (CAZy) with HMMER v3.3 (20), and KEGG with GhostKOALA (21). The 2,422 species-like OTU representatives were further annotated by AntiSMASH v6.1 (22) to predict secondary metabolite biosynthetic gene clusters (BGCs).

A gene tree was constructed to distinguish *rbcL* encoding the large chain of ribulose-bisphosphate carboxylase from its paralog involved in nucleotide consumption (23). Briefly, the *rbcL*-like sequences were extracted from the TPLM genomes based on KEGG Ortholog (KO) annotation (K01601) and aligned against reference sequences reported previously (24) at the amino acid sequence level using MAFFT v7.455 (25). The phylogenetic tree of *rbcL* was then constructed using IQ-Tree v2.2.0 (26) with LG+I+G model and visualized using iTOL (27). More gene trees were constructed similarly to distinguish *amoA* genes for nitrification from *pmoA* genes for methane oxidation (28), high-affinity *amtB* genes for ammonium

transport from those with low-affinity (29), and reductive *dsrA* genes for sulfite reduction from oxidative *rdsrA* genes for sulfide oxidation (30).

Taxonomic inference and phylogenetic analysis

Taxonomic annotation of MAGs was performed using GTDB-Tk (31) with the database version RS202 and toolkit version v1.7.0. To validate the phylogenetic position of the 8,271 MAGs, archaeal and bacterial phylogenomic trees were reconstructed based on GTDB alignments of the 8,271 MAGs and GTDB reference genomes using FastTree v2.1.11 (32). The archaeal and bacterial trees each were rooted according to the GTDB database and visualized using iTOL (27). To assess the novelty of TPLM genomes, representative genomes from TPLM atlas, GTDB database, and the Tibetan Plateau Microbial Catalog (TPMC) (33) were combined to build a phylogenomic tree using FastTree v2.2.1 (32). The phylogenetic diversity (total branch length spanned by reference genomes in the GTDB database) and gain (additional branch length contributed by TPLM genomes) were calculated using custom scripts.

Recruitment analysis

Recruitment analysis was performed by mapping clean reads to the 2,422 species-like OTU representatives using bowtie2 v2.2.5 (34) with options '--very-sensitive-local'. Genome coverage and sequencing depth of species-like OTUs were estimated using SAMtools (35). The relative abundance of OTUs was then calculated using custom scripts in the form of Transcripts Per Million (TPM), a measure that normalizes the read count for each OTU by genome size and sequencing depth (36).

Specifically, recruitment analysis was reperformed against all 8,271 MAGs to calculate the proportion of reads that were mapped by the TPLM genome atlas.

Statistical analysis of genomic features

Genomic features of microbiomes in different salinity groups of lakes were investigated, including estimated genome size, coding density, GC content, carbon/nitrogen atoms per residue side chain (C/N-ARSC), number of CAZY genes per Mb, and optimal growth temperature. Assembled genome size, coding density, and GC content were summarized using CheckM v1.1.3 (12). The estimated genome size was then calculated as the assembled genome size divided by the sum of genome completeness and contamination (16). C/N-ARSC and the number of CAZY genes per Mb were summarized using custom scripts. The optimal growth temperature was predicted using Tome v1.0.0 (37).

The aforementioned genomic features were estimated for the 2,422 genomes each representing a species-like OTU. The value of a genomic feature of the microbiome in each lake sample was calculated as the mean value of the genomic feature across the 2,422 OTUs weighted by the relative abundance of each OTU. Statistical analysis was performed using R package ‘ggpubr’ v0.4.0 (https://rpkgs.datanovia.com/ggpubr).

Identification of functional genes enriched in different salinity groups of lakes

Metabolic differentiation between freshwater lakes, brackish lakes, and saline lakes on the Tibetan Plateau was predicted by assessing the relative abundance of functional genes. Assuming that a KO exhibit similar gene length in different organisms, the TPM of a KEGG Orthology (KO) in a given lake sample was

calculated as the sum of the TPM across the 2,422 OTUs each multiplied by the corresponding KO copy number within that OTU (38). Subsequently, the relative abundance of each KO in this sample was normalized by dividing the TPM of the KO by the average TPM of the 27 universal single-copy KOs (39). Enrichment of KOs between freshwater and saline lakes was identified using Welch's t-test and *p* values were corrected using FDR method. The relative abundance of BGC categories was compared using STAMP v2.1.3 (40) and *p* values were corrected using the FDR method.

Microbial community profiling

Taxonomic diversity was assessed at the genus level from metagenomic clean reads based on the 14 marker genes (41) using SingleM (https://github.com/wwood/singlem), which provides enhanced taxonomic classification with improved resolution and reduced amplification bias compared to the traditional methods based on 16S rRNA gene amplicon sequences. Functional diversity was evaluated based on the relative abundance of KOs mentioned above. Shannon diversity within a sample and Bray-Curtis dissimilarities between samples were calculated using R package ‘vegan’ v2.5 (https://github.com/vegandevs/vegan). The relationship of taxonomic and functional profiles with environmental factors was inferred using the partial Mantel test with 1,000 permutations and Bonferroni correction (42). The correlation between environmental factors was inferred using Spearman correlation with Bonferroni correction. The proportion of the variation of taxonomic and functional community structure that can be explained by ecological

factors were estimated using R package ‘vegan’. Enrichment of major phyla and major classes within the phylum Proteobacteria in freshwater or saline lakes was identified using STAMP v2.1.3 (40) and *p* values were corrected using the FDR method.

Evolutionary analyses

We conducted the assessments of intraspecific population genetic parameters for each species-like OTU in each of the 169 samples. To ensure robustness in downstream evolutionary analyses, only OTUs with a genome coverage of >80% and sequencing depth of >5 in the metagenomic recruitment were retained. To compare the genetic diversity within and selection effectiveness on populations between freshwater lakes, brackish lakes, and saline lakes, genome-wide nucleotide diversity (π), single-nucleotide variant (SNV) per Mb, and ratio of nonsynonymous versus synonymous variants (*pN/pS*) were estimated using inStrain v1.6.3 (43). To examine the impact of recombination, the relative rate (ρ/θ) of recombination to mutation were predicted using mcorr (44) based on recruitment results without assembly and using ClonalFrameML v1.12 (45) based on metagenomic assembled contigs following a previous study (46), respectively. The relative effect (*r/m*) of recombination to mutation was estimated using ClonalFrameML. Statistical analysis was performed using R package ‘ggpubr’ v0.4.0 (https://rpkgs.datanovia.com/ggpubr). The phylogenetic null model analysis was performed following previous study (47).

References

1. APHA/AWWA/WEF. Standard Methods for the Examination of Water and Wastewater Stand Methods; 2017.

2. Gong Y, Tang X, Shao K, Hu Y, Gao G. Dynamics of bacterial abundance and the related environmental factors in large shallow eutrophic Lake Taihu. *Journal of Freshwater Ecology* 2017; 32(1):133–45.

3. Bolger AM, Lohse M, Usadel B. Trimmomatic: a flexible trimmer for Illumina sequence data. *Bioinformatics* 2014; 30(15):2114–20.

4. Li H, Durbin R. Fast and accurate short read alignment with Burrows-Wheeler transform. *Bioinformatics* 2009; 25(14):1754–60.

5. Smith MW, Herfort L, Rivers AR, Simon HM. Genomic Signatures for Sedimentary Microbial Utilization of Phytoplankton Detritus in a Fast-Flowing Estuary. *Front Microbiol* 2019; 10:2475.

6. Li D, Liu C-M, Luo R, Sadakane K, Lam T-W. MEGAHIT: an ultra-fast single-node solution for large and complex metagenomics assembly via succinct de Bruijn graph. *Bioinformatics* 2015; 31(10):1674–6.

7. Uritskiy GV, DiRuggiero J, Taylor J. MetaWRAP-a flexible pipeline for genome-resolved metagenomic data analysis. *Microbiome* 2018; 6(1):158.

8. Kang D, Li F, Kirton ES, Thomas A, Egan RS, An H et al. MetaBAT 2: an adaptive binning algorithm for robust and efficient genome reconstruction from metagenome assemblies. *PeerJ* 2019; 7:e7359.

9. Alneberg J, Bjarnason BS, Bruijn I de, Schirmer M, Quick J, Ijaz UZ et al. Binning metagenomic contigs by coverage and composition. *Nat. Methods* 2014; 11(11):1144–6.

10. Wu Y-W, Simmons BA, Singer SW. MaxBin 2.0: an automated binning algorithm to recover genomes from multiple metagenomic datasets. *Bioinformatics* 2016; 32(4):605–7.

11. Sieber CMK, Probst AJ, Sharrar A, Thomas BC, Hess M, Tringe SG et al. Recovery of genomes from metagenomes via a dereplication, aggregation and scoring strategy. *Nat Microbiol* 2018; 3(7):836–43.

12. Parks DH, Imelfort M, Skennerton CT, Hugenholtz P, Tyson GW. CheckM: assessing the quality of microbial genomes recovered from isolates, single cells, and metagenomes. *Genome Res.* 2015; 25(7):1043–55.

13. Bowers RM, Kyrpides NC, Stepanauskas R, Harmon-Smith M, Doud D, Reddy TBK et al. Minimum information about a single amplified genome (MISAG) and a metagenome-assembled genome (MIMAG) of bacteria and archaea. *Nat. Biotechnol.* 2017; 35(8):725–31.

14. Olm MR, Brown CT, Brooks B, Banfield JF. dRep: a tool for fast and accurate

genomic comparisons that enables improved genome recovery from metagenomes through de-replication. *ISME J* 2017; 11(12):2864–8.

15. Jain C, Rodriguez-R LM, Phillippy AM, Konstantinidis KT, Aluru S. High throughput ANI analysis of 90K prokaryotic genomes reveals clear species boundaries. *Nat. Commun.* 2018; 9(1):5114.

16. Parks DH, Rinke C, Chuvochina M, Chaumeil P-A, Woodcroft BJ, Evans PN et al. Recovery of nearly 8,000 metagenome-assembled genomes substantially expands the tree of life. *Nat Microbiol* 2017; 2(11):1533–42.

17. Hyatt D, Chen G-L, Locascio PF, Land ML, Larimer FW, Hauser LJ. Prodigal: prokaryotic gene recognition and translation initiation site identification. *BMC Bioinformatics* 2010; 11:119.

18. Mirdita M, Steinegger M, Breitwieser F, Söding J, Levy Karin E. Fast and sensitive taxonomic assignment to metagenomic contigs. *Bioinformatics* 2021; 37(18):3029–31.

19. Huerta-Cepas J, Szklarczyk D, Heller D, Hernández-Plaza A, Forslund SK, Cook H et al. eggNOG 5.0: a hierarchical, functionally and phylogenetically annotated orthology resource based on 5090 organisms and 2502 viruses. *Nucleic Acids Res.* 2018; 47(D1):D309-D314.

20. Drula E, Garron M-L, Dogan S, Lombard V, Henrissat B, Terrapon N. The carbohydrate-active enzyme database: functions and literature. *Nucleic Acids Res.* 2022; 50(D1):D571-D577.

21. Kanehisa M, Goto S. KEGG: kyoto encyclopedia of genes and genomes. *Nucleic Acids Res.* 2000; 28(1):27–30.

22. Blin K, Shaw S, Kloosterman AM, Charlop-Powers Z, van Wezel GP, Medema MH et al. antiSMASH 6.0: improving cluster detection and comparison capabilities. *Nucleic Acids Res.* 2021; 49(W1):W29-W35.

23. Aono R, Sato T, Imanaka T, Atomi H. A pentose bisphosphate pathway for nucleoside degradation in Archaea. *Nat Chem. Biol.* 2015; 11(5):355–60.

24. Jaffe AL, Castelle CJ, Dupont CL, Banfield JF. Lateral Gene Transfer Shapes the Distribution of RuBisCO among Candidate Phyla Radiation Bacteria and DPANN Archaea. *Mol. Biol. Evol.* 2019; 36(3):435–46.

25. Katoh K, Standley DM. MAFFT multiple sequence alignment software version 7: improvements in performance and usability. *Mol. Biol. Evol.* 2013; 30(4):772–80.

26. Minh BQ, Schmidt HA, Chernomor O, Schrempf D, Woodhams MD, Haeseler A von et al. IQ-TREE 2: New Models and Efficient Methods for Phylogenetic Inference in the Genomic Era. *Mol. Biol. Evol.* 2020; 37(5):1530–4.

27. Letunic I, Bork P. Interactive Tree Of Life (iTOL) v5: an online tool for phylogenetic tree display and annotation. *Nucleic Acids Res.* 2021; 49(W1):W293-W296.

28. Khadka R, Clothier L, Wang L, Lim CK, Klotz MG, Dunfield PF. Evolutionary History of Copper Membrane Monooxygenases. *Front Microbiol* 2018; 9:2493.

29. McDonald TR, Ward JM. Evolution of Electrogenic Ammonium Transporters (AMTs). *Front. Plant Sci.* 2016; 7:352.

30. Müller AL, Kjeldsen KU, Rattei T, Pester M, Loy A. Phylogenetic and environmental diversity of DsrAB-type dissimilatory (bi)sulfite reductases. *ISME J* 2015; 9(5):1152–65.

31. Chaumeil P-A, Mussig AJ, Hugenholtz P, Parks DH. GTDB-Tk: a toolkit to classify genomes with the Genome Taxonomy Database. *Bioinformatics* 2019:1925–7.

32. Price MN, Dehal PS, Arkin AP. FastTree 2--approximately maximum-likelihood trees for large alignments. *PLoS ONE* 2010; 5(3):e9490.

33. Mingyue Cheng, Shuai Luo, Peng Zhang, Guangzhou Xiong, Kai Chen, Chuanqi Jiang et al. A genome and gene catalog of the aquatic microbiomes of the Tibetan Plateau 2024.

34. Langmead B, Salzberg SL. Fast gapped-read alignment with Bowtie 2. *Nat. Methods* 2012; 9(4):357–9.

35. Danecek P, Bonfield JK, Liddle J, Marshall J, Ohan V, Pollard MO et al. Twelve years of SAMtools and BCFtools. *Gigascience* 2021; 10(2):giab008.

36. Wagner GP, Kin K, Lynch VJ. Measurement of mRNA abundance using RNA-seq data: RPKM measure is inconsistent among samples. *Theory Biosci.* 2012; 131(4):281–5.

37. Gang Li, Kersten S. Rabe, Jens Nielsen, and Martin K. M. Engqvist. Machine Learning Applied to Predicting Microorganism Growth Temperatures and Enzyme Catalytic Optima. *ACS Synth. Biol.* 2019; 8(6):1411–20.

38. Zhou Y-L, Mara P, Cui G-J, Edgcomb VP, Wang Y. Microbiomes in the Challenger Deep slope and bottom-axis sediments. *Nat. Commun.* 2022; 13(1):1515.

39. Coleman GA, Davín AA, Mahendrarajah TA, Szánthó LL, Spang A, Hugenholtz P et al. A rooted phylogeny resolves early bacterial evolution. *Science* 2021; 372(6542).

40. Parks DH, Tyson GW, Hugenholtz P, Beiko RG. STAMP: statistical analysis of taxonomic and functional profiles. *Bioinformatics* 2014; 30(21):3123–4.

41. Woodcroft BJ, Singleton CM, Boyd JA, Evans PN, Emerson JB, Zayed AAF et al. Genome-centric view of carbon processing in thawing permafrost. *Nature* 2018; 560(7716):49–54.

42. Salazar G, Paoli L, Alberti A, Huerta-Cepas J, Ruscheweyh H-J, Cuenca M et al. Gene expression changes and community turnover differentially shape the global ocean metatranscriptome. *Cell* 2019; 179(5):1068-1083.e21.

43. Olm MR, Crits-Christoph A, Bouma-Gregson K, Firek BA, Morowitz MJ, Banfield JF. inStrain profiles population microdiversity from metagenomic data and

sensitively detects shared microbial strains. *Nat. Biotechnol.* 2021; 39(6):727–36.

44. Lin M, Kussell E. Inferring bacterial recombination rates from large-scale sequencing datasets. *Nat. Methods* 2019; 16(2):199–204.

45. Didelot X, Wilson DJ. ClonalFrameML: efficient inference of recombination in whole bacterial genomes. *PLoS Comput. Biol.* 2015; 11(2):e1004041.

46. Ngugi DK, Salcher MM, Andrei A-S, Ghai R, Klotz F, Chiriac M-C et al. Postglacial adaptations enabled colonization and quasi-clonal dispersal of ammonia-oxidizing archaea in modern European large lakes. *Sci Adv* 2023; 9(5):eadc9392.

47. Ning D, Yuan M, Wu L, Zhang Y, Guo X, Zhou X et al. A quantitative framework reveals ecological drivers of grassland microbial community assembly in response to warming. *Nat Commun* 2020; 11(1):4717.

Fig. S1. Comparison of ecological factors across the Tibetan Plateau lakes using violin plot. Freshwater, brackish, and saline lakes are colored in green, gray, and blue, respectively. The *p* values of <0.001 based on Welch's t-test between different types of lakes are colored in red. The concentrations of nutrients and ions are logarithmically transformed for better visualization.

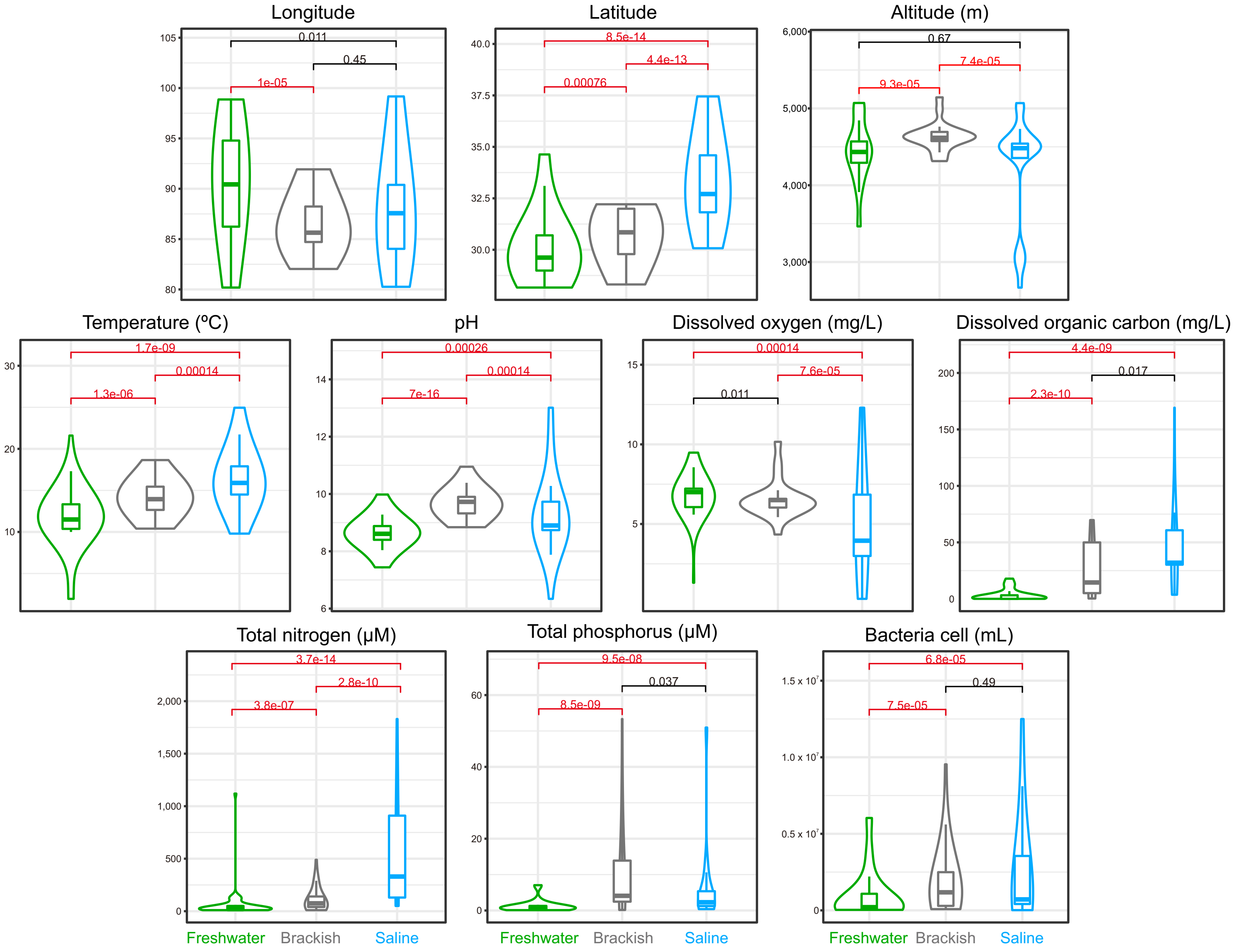

Fig. S2. Taxonomic composition and microbial diversity of the Tibetan Plateau lakes. Samples are arranged along salinity gradient and those from freshwater, brackish, and saline lakes are colored in green, gray, and blue, respectively. Taxonomic analyses are performed based on 14 marker genes using SingleM. **(A)** The relative abundance of major phyla and major classes within the phylum Proteobacteria. The taxonomic groups enriched in freshwater and saline lakes (*p* value < 0.01 based on Welch's t-test) are colored in green and blue, respectively. (B) Microbial diversity at taxonomic and functional levels. The Shannon index at functional level is calculated based on 15,943 functional genes.

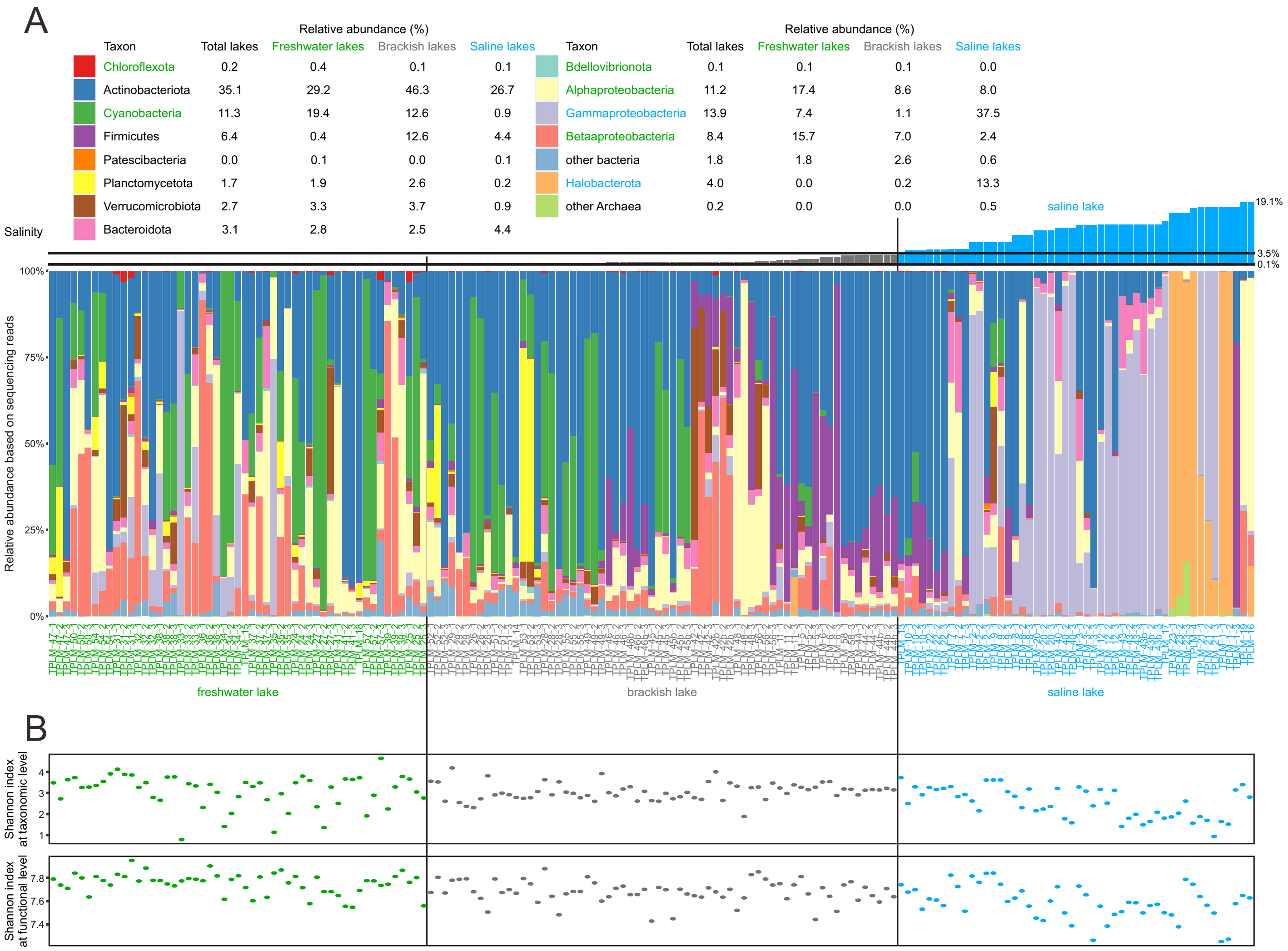

Fig. S3. Community variations at taxonomic and functional levels. This figure parallels Fig. 1B, but groups samples based on filter pore sizes. Community similarities between different types of Tibet lakes are assessed using ANOSIM analysis with significance of >0.05 for all estimations.

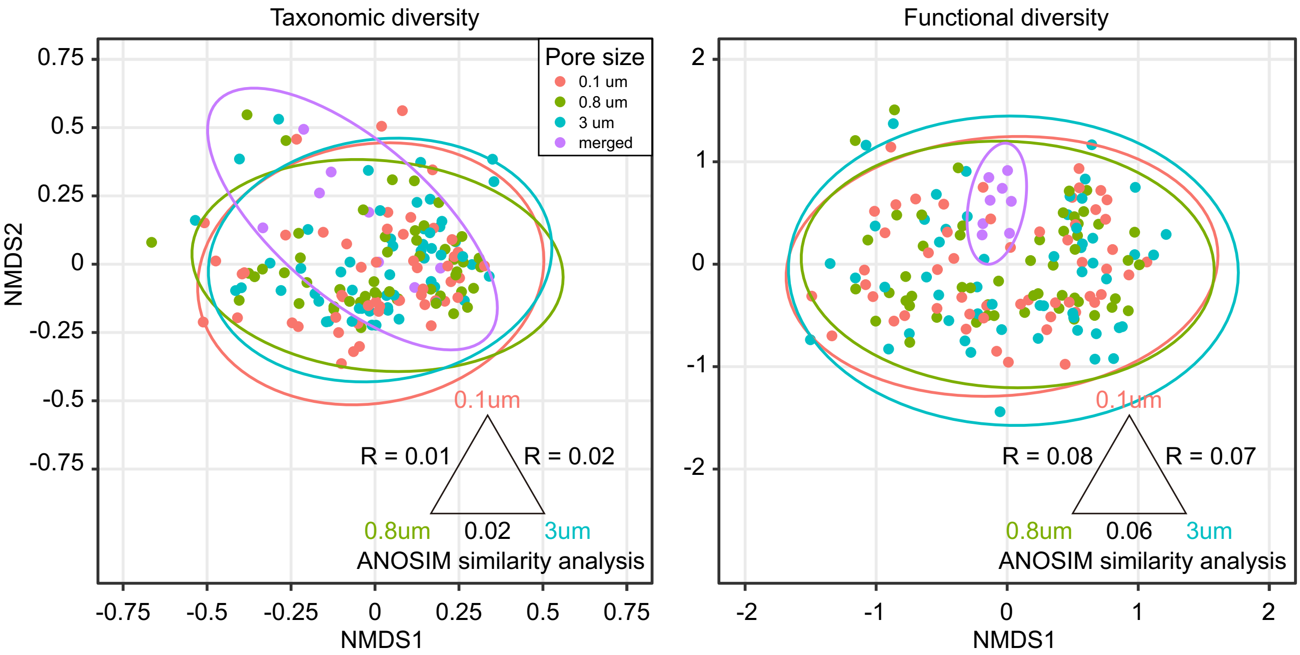

Fig. S4. Microbial profiles and ecological driving factors. Pearson's correlation coefficients are marked with a color gradient in cells with adjusted p values of <0.01. Nonsignificant correlations are marked using crosses. **(A)** Correlation of taxonomic and functional profiles with ecological factors. The correlation analysis is performed using partial Mantel tests with 1,000 permutations and Bonferroni correction. **(B)** Pairwise comparisons of ecological factors across the Tibetan Plateau lakes.

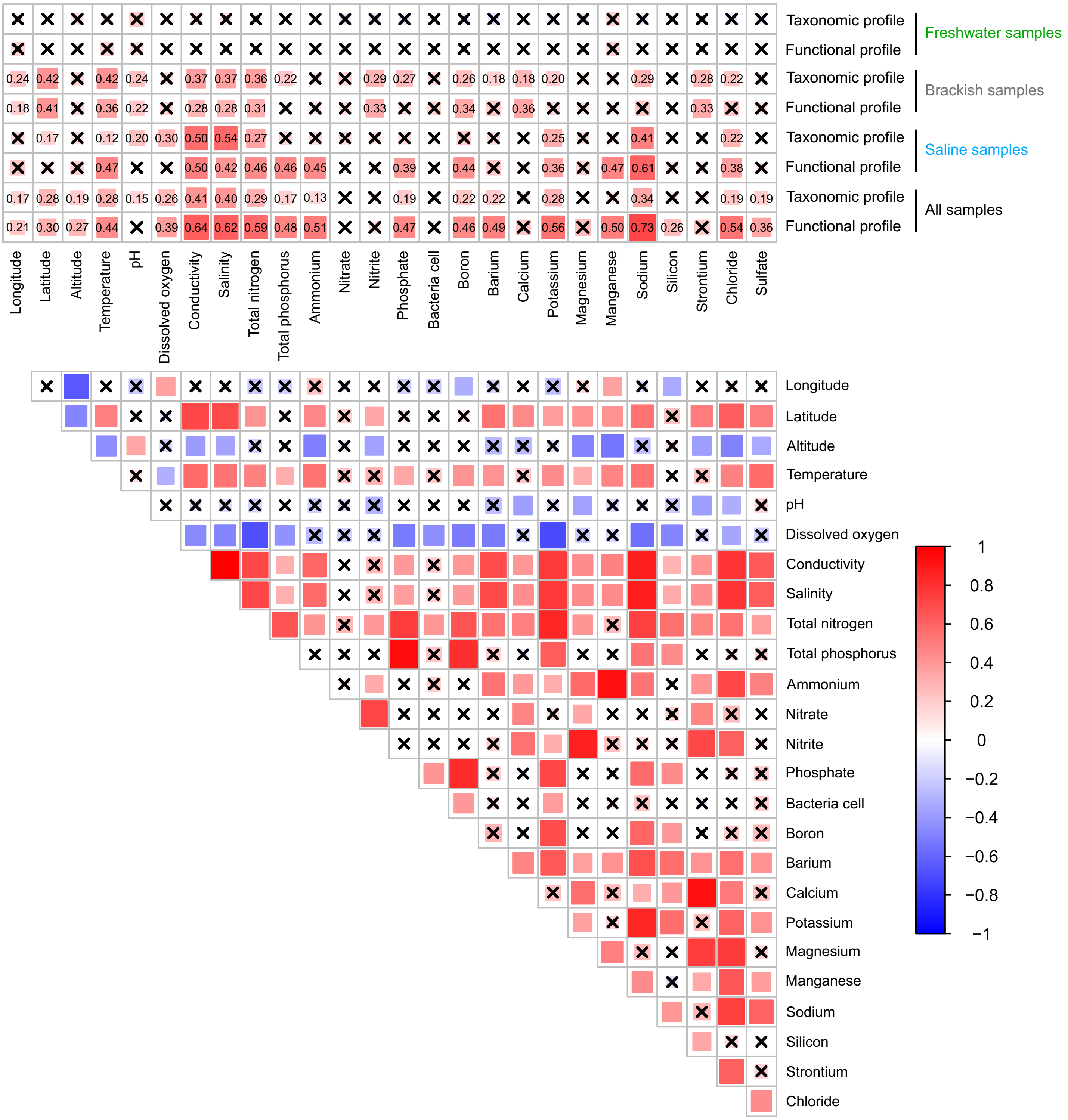

Fig. S5. Genomic novelty compared to TPMC database. These figures parallel Fig. 2. **(A)** Phylogenetic diversity (PD) gain in comparison to a combination of GTDB reference genomes (RS202) and TPMC representative genomes. **(B)** Phylogenetic tree showcasing 2,422 TPLM species-like OTUs and 13,145 TPMC reference genomes. TPLM genomes with ANI above or below 95% compared to TPMC are marked with blue and red dots, respectively.

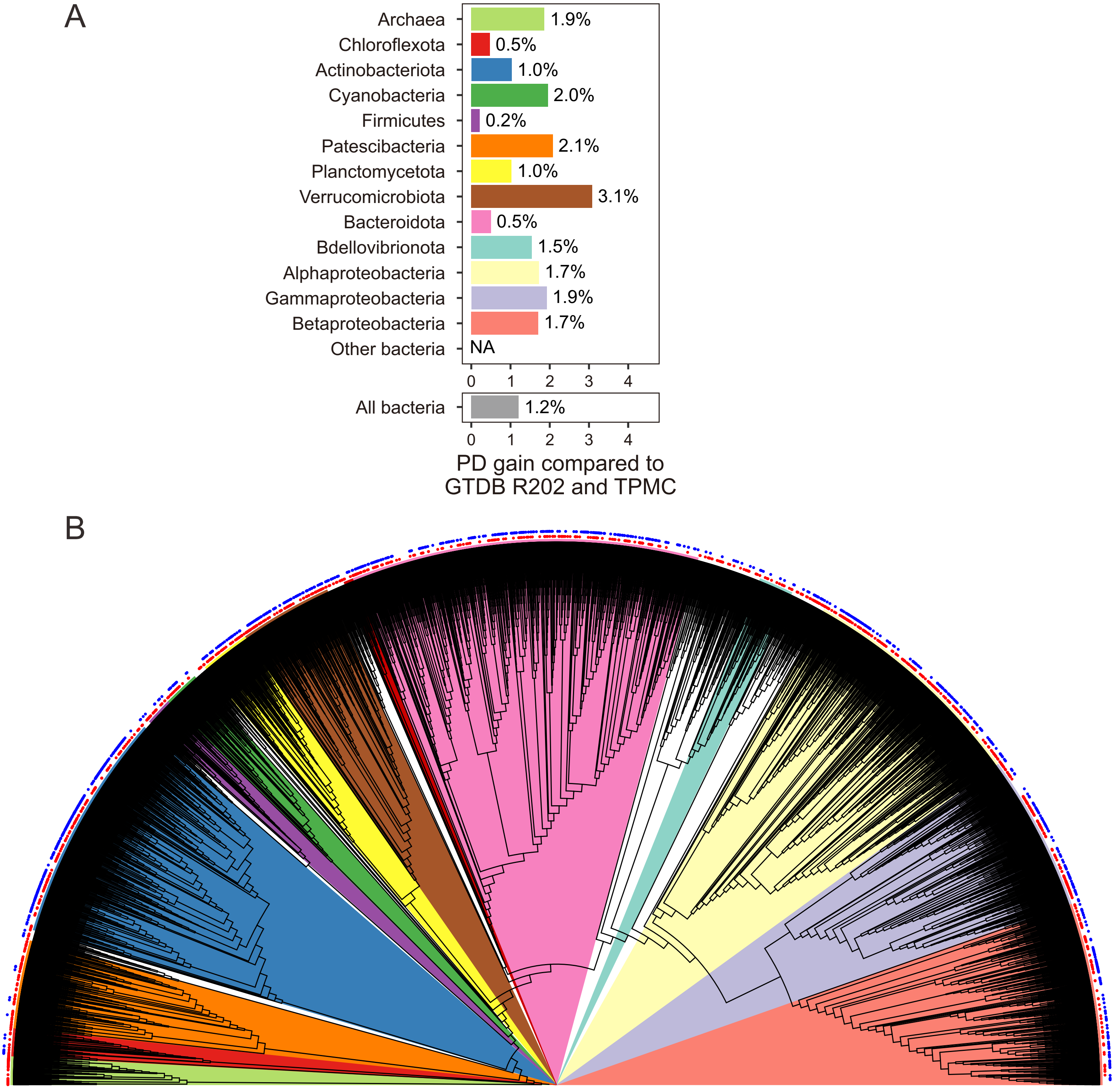

Fig. S6. BGC distribution in the TPLM database. **(A)** The number of BGCs across major phyla and major classes within the phylum Proteobacteria. The BGCs were predicted from the 2,422 species-like OTU representatives using AntiSMASH v6.1. The BGC categories are shown using different colors.
(B) Comparison of BGC categories between freshwater lake and saline lake microbiomes. The enrichment analysis is performed between freshwater and saline lakes using STAMP, and categories significantly enriched in freshwater or saline lake microbiomes (FDR adjusted *p* value < 1) are colored in green and blue, respectively. Categories without enrichment patterns are colored in black.

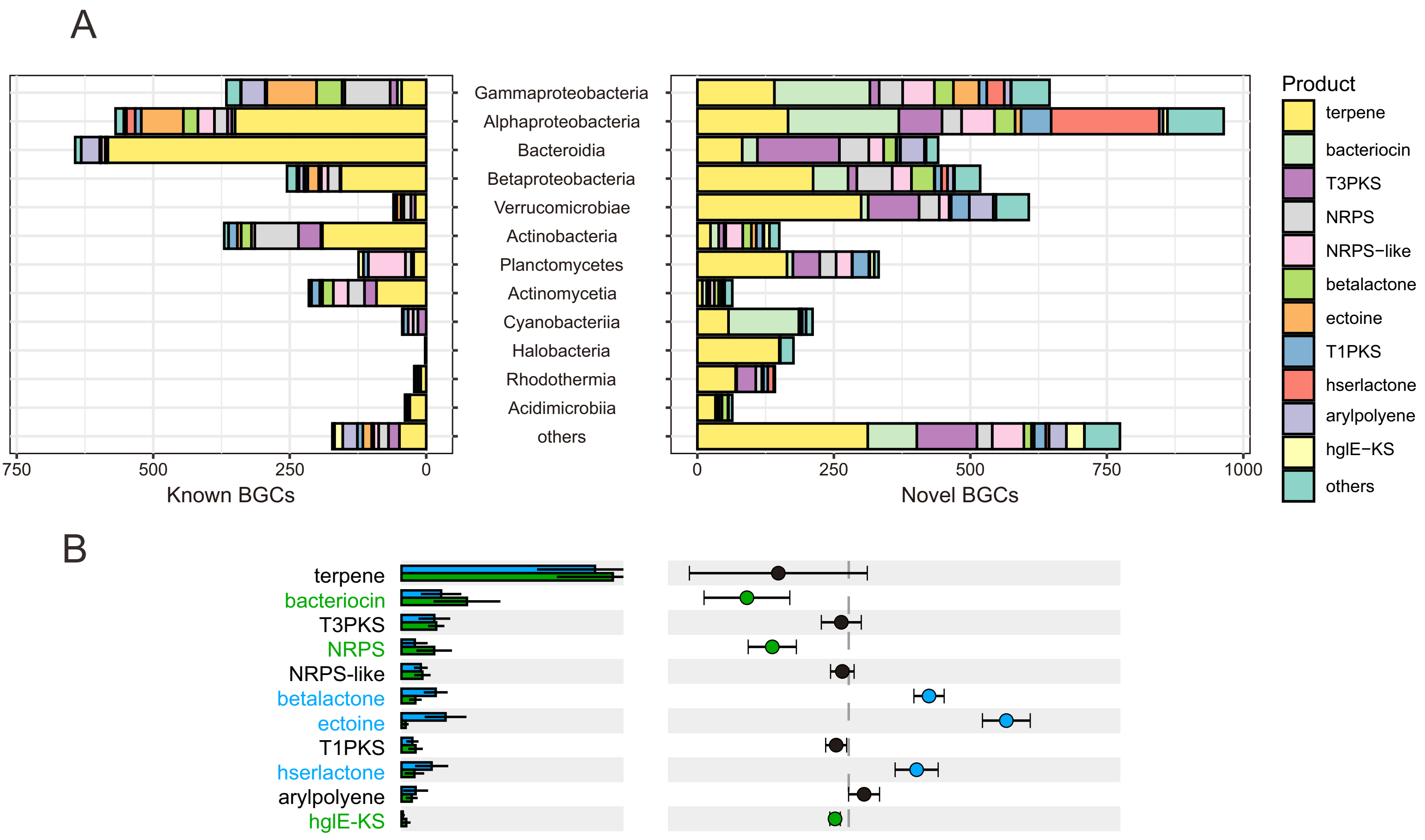

Fig. S7. Comparison of genomic and evolutionary features using violin plots. Freshwater, brackish, and saline colored in green, gray, and blue, respectively. The p values of <0.001 based on Welch's t-test between different types of lakes are colored in red. CAZy, carbohydrate-active enzymes; C-ARSC, carbon atoms per residue side chain; N-ARSC, nitrogen atoms per residue side chain.

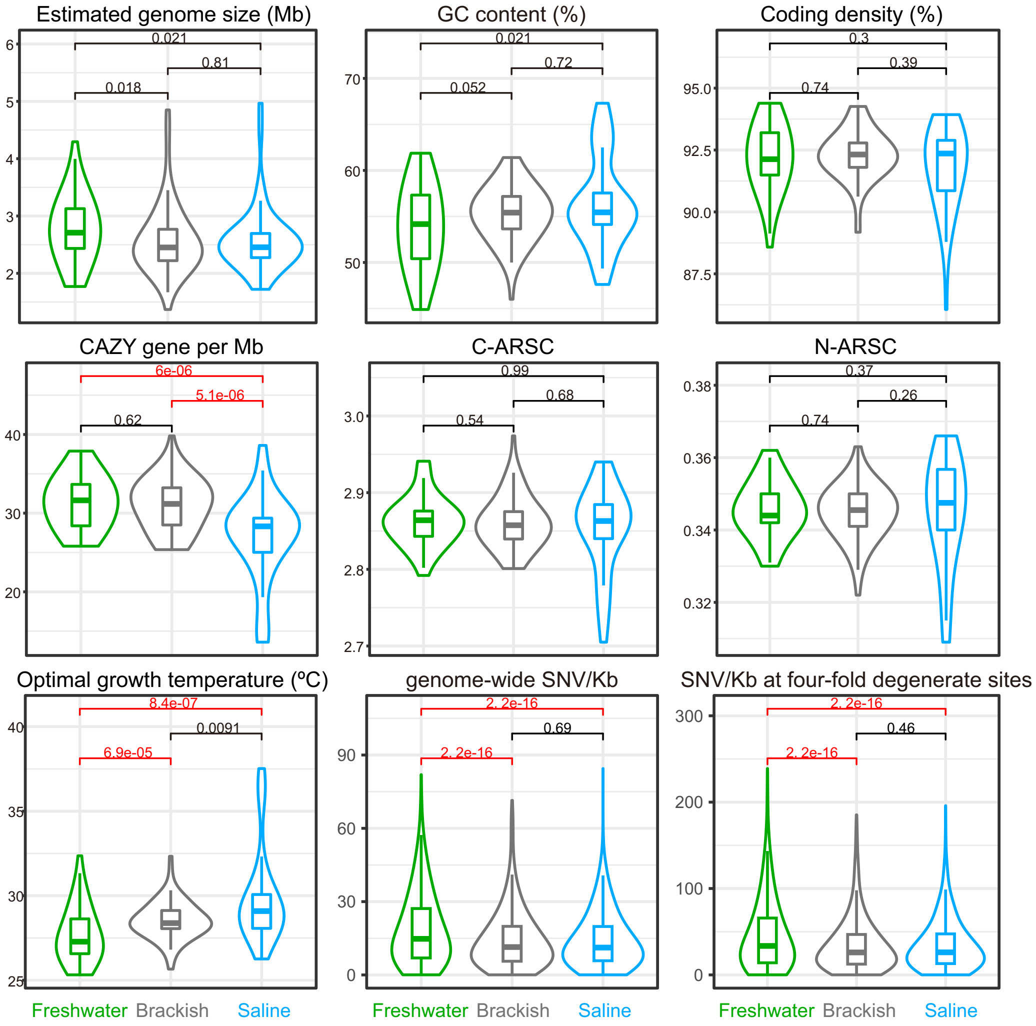

Fig. S8. Phylogenetic tree for selected functional genes. The maximum likelihood trees are built using IQ-Tree with LG+I+G model. Functional genes extracted from TPLM MAGs are marked using red dots. Nodes with bootstrap values of >95% are marked with black dots in the phylogeny. Reference sequences and category classification are used following previous studies for *rbcL* genes **(A)**, *amoA* and *pmoA* genes **(B)**, *amt* genes **(C)**, and *dsrA* and *rdsrA* genes **(D)**, respectively.

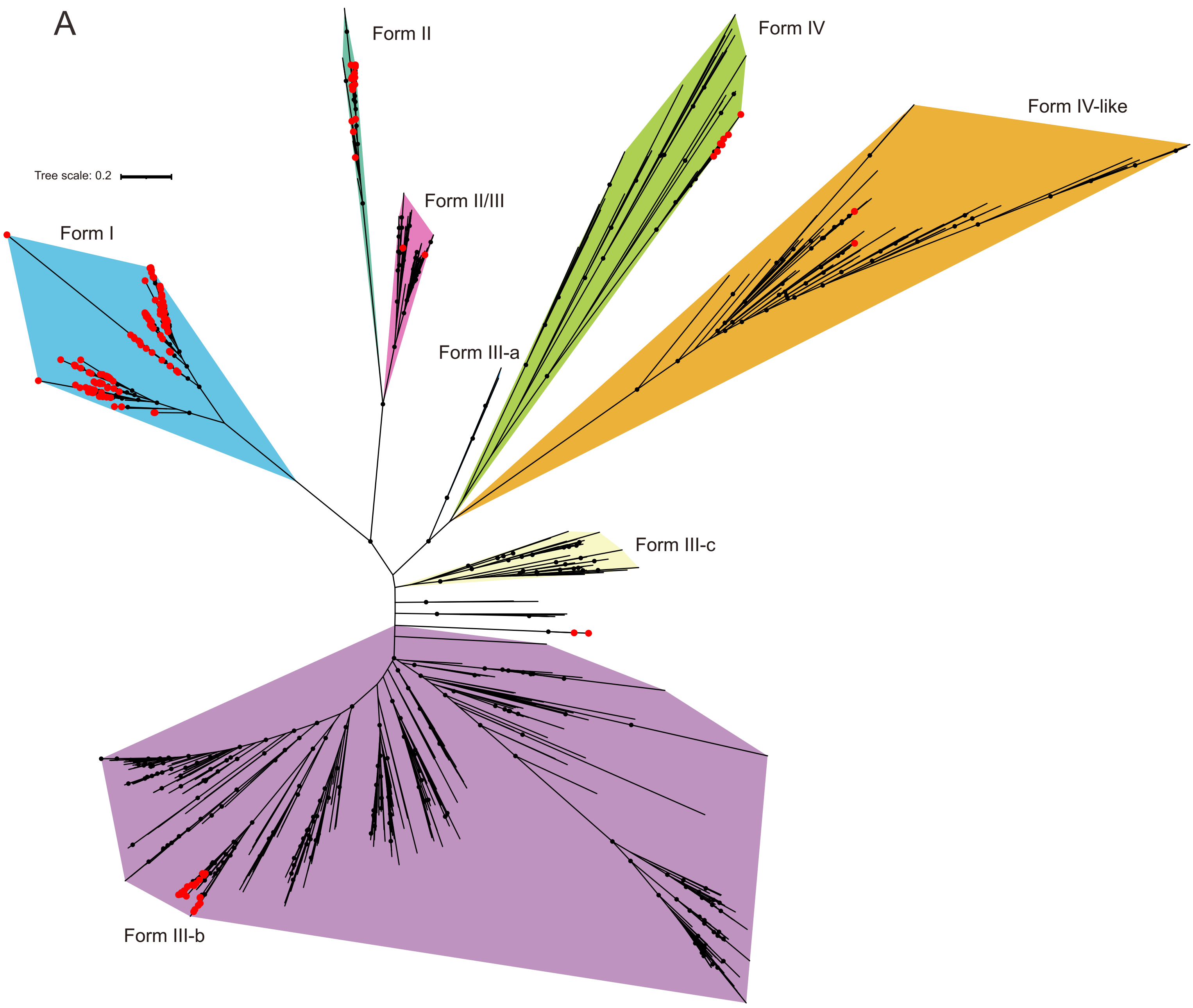

B

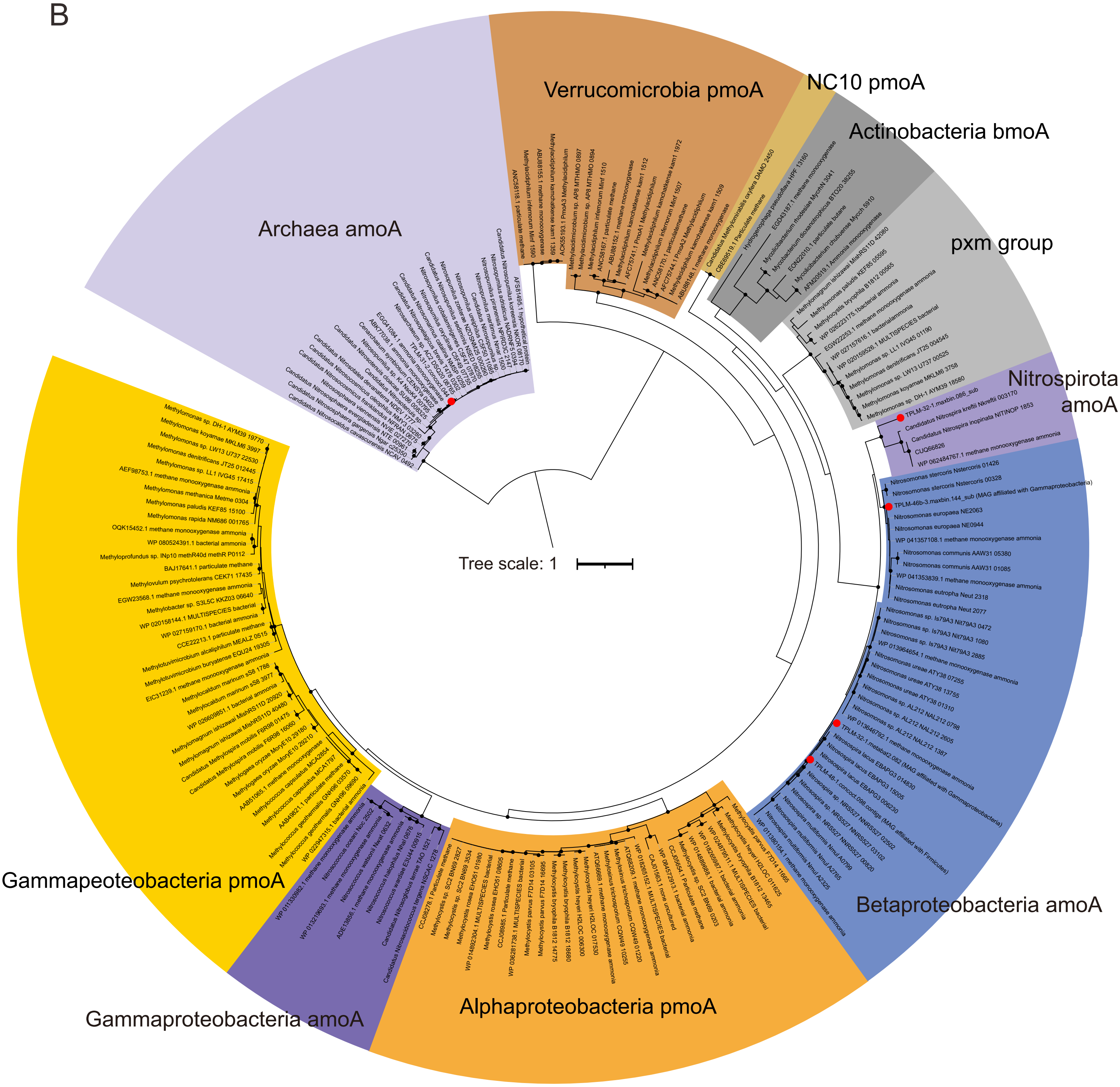
Verrucomicrobia pmoA
NC10 pmoA
Actinobacteria bmoA
Archaea amoA
pxm group
Nitrospirota amoA
Betaproteobacteria amoA
Alphaproteobacteria pmoA
Gammaproteobacteria amoA
Gammapeoteobacteria pmoA
Tree scale: 1

C

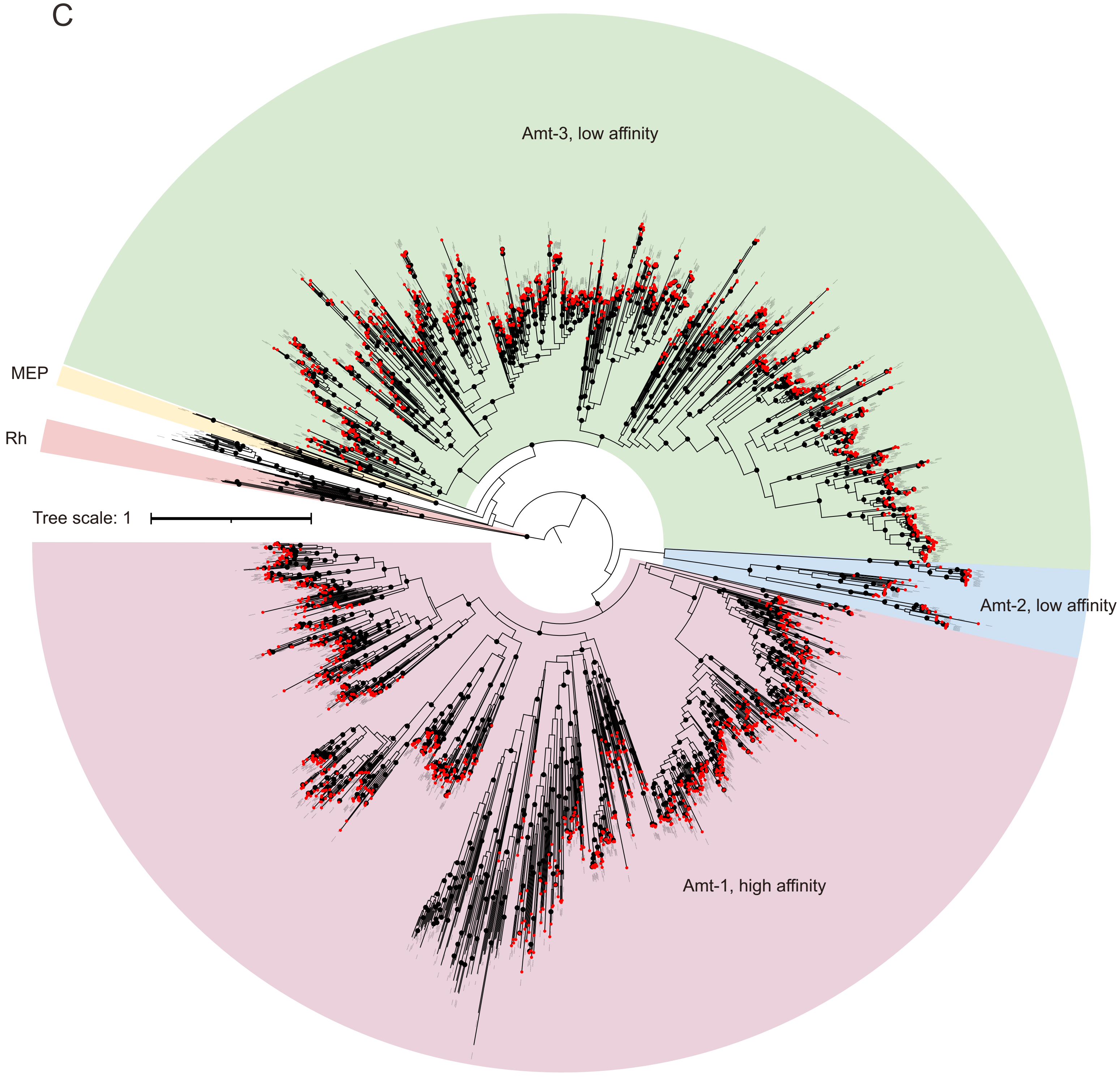
Amt-3, low affinity
MEP
Rh
Tree scale: 1
Amt-2, low affinity
Amt-1, high affinity

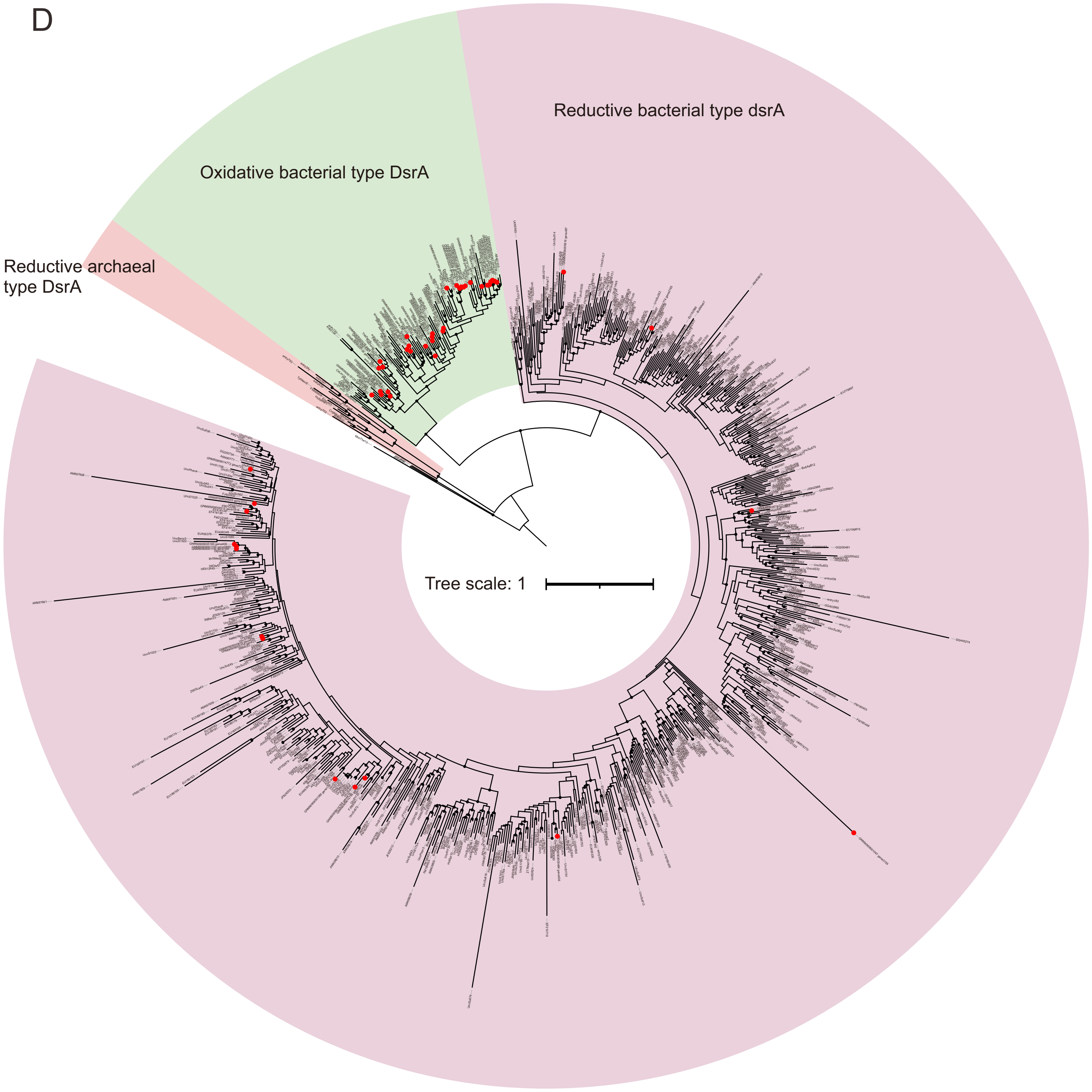
D
Reductive bacterial type dsrA
Oxidative bacterial type DsrA
Reductive archaeal type DsrA
Tree scale: 1

Fig. S9. The correlation of *rbcL*, *psaA,* and *psbA* genes in lake microbiomes. The relative abundance of a specific gene was calculated using the Transcript Per Million (TPM) of this gene divided by the average TPM of 27 universal single-copy KOs. The correlations of the Calvin cycle marker gene (type I/II *rbcL*) with the photosystem marker genes (*psaA,* and *psbA*) are estimated. Freshwater, brackish, and saline lakes are colored in green, gray, and blue, respectively. The R^2 values and significances are given in each panel.

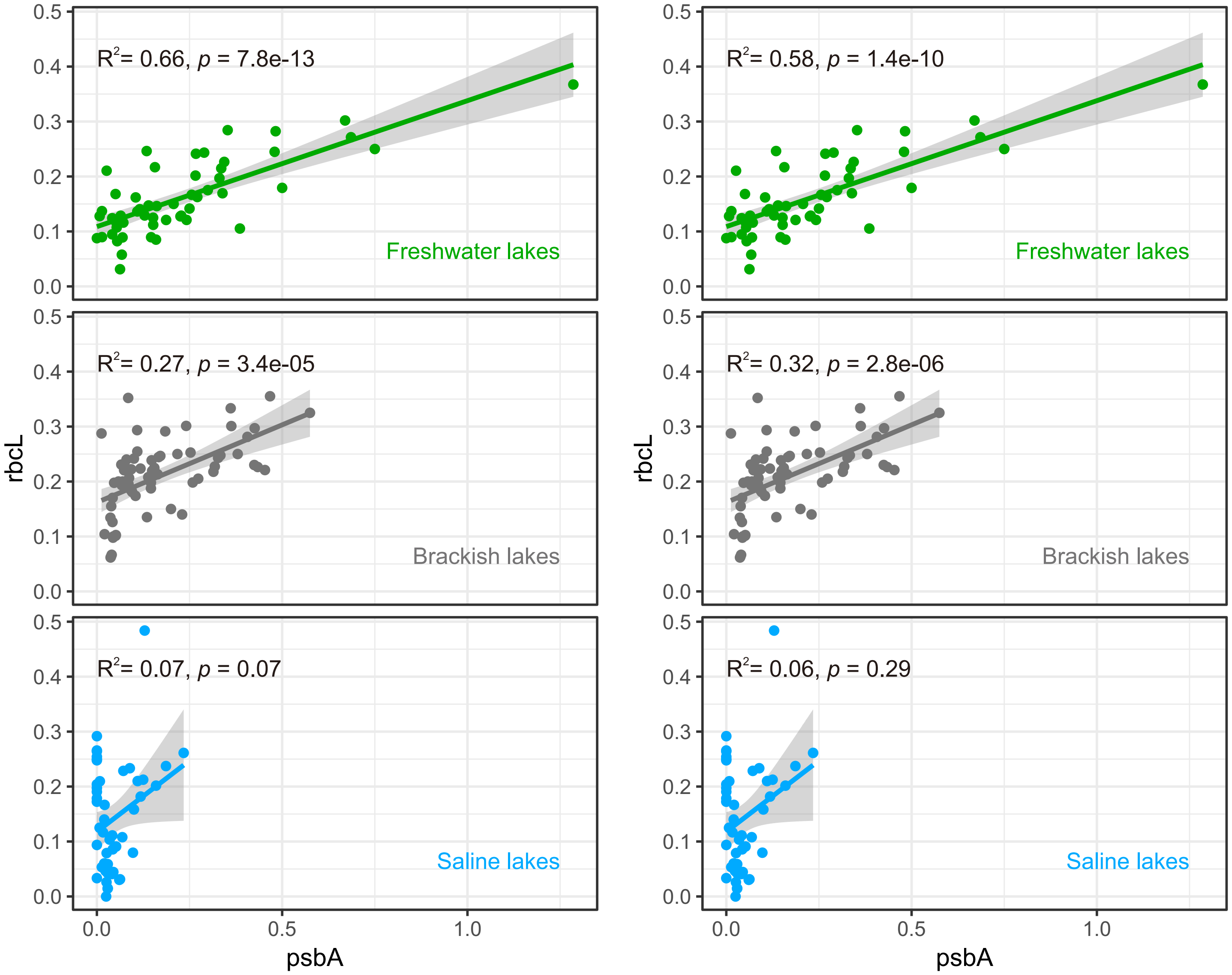

Fig. S10. The contribution of the ecological processes that determined community assembly in studied Tibet lakes. The effects of homogenizing dispersal and drift are found to be negligible across all lakes and are therefore omitted for clarity. Freshwater, brackish, and saline lakes are colored in green, gray, and blue, respectively.

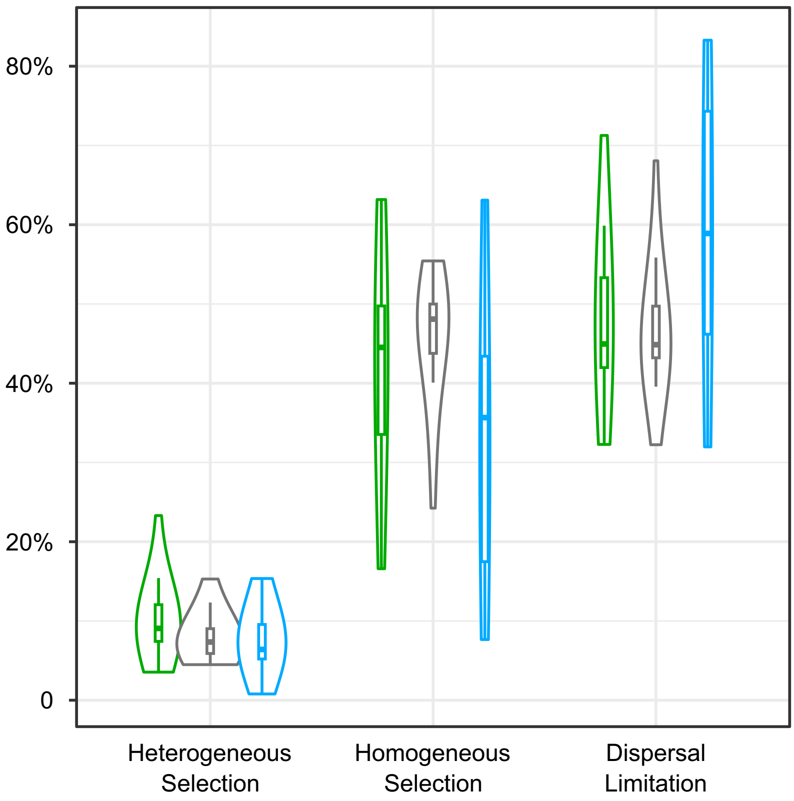

Supplement: Supplementary file 2 — Supplementary Material 1. [file 40168_2024_1979_MOESM1_ESM.pdf]
